# MAP-Kinase Regulated Cytosolic Phospholipase A2 Activity Is Essential for Production of Infectious Hepatitis C Virus Particles

**DOI:** 10.1371/journal.ppat.1002829

**Published:** 2012-07-26

**Authors:** Nicolas Menzel, Wolfgang Fischl, Kathrin Hueging, Dorothea Bankwitz, Anne Frentzen, Sibylle Haid, Juliane Gentzsch, Lars Kaderali, Ralf Bartenschlager, Thomas Pietschmann

**Affiliations:** 1 Division of Experimental Virology, TWINCORE, Centre for Experimental and Clinical Infection Research; a joint venture between the Medical School Hannover (MHH) and the Helmholtz Centre for Infection Research (HZI), Hannover, Germany; 2 Department of Infectious Diseases, Molecular Virology, University Hospital Heidelberg, Heidelberg, Germany; 3 Institute for Medical Informatics and Biometry (IMB), Medical Faculty Carl Gustav Carus, Dresden University of Technology, Dresden, Germany; Yale University, United States of America

## Abstract

Hepatitis C virus (HCV) has infected around 160 million individuals. Current therapies have limited efficacy and are fraught with side effects. To identify cellular HCV dependency factors, possible therapeutic targets, we manipulated signaling cascades with pathway-specific inhibitors. Using this approach we identified the MAPK/ERK regulated, cytosolic, calcium-dependent, group IVA phospholipase A2 (PLA2G4A) as a novel HCV dependency factor. Inhibition of PLA2G4A activity reduced core protein abundance at lipid droplets, core envelopment and secretion of particles. Moreover, released particles displayed aberrant protein composition and were 100-fold less infectious. Exogenous addition of arachidonic acid, the cleavage product of PLA2G4A-catalyzed lipolysis, but not other related poly-unsaturated fatty acids restored infectivity. Strikingly, production of infectious Dengue virus, a relative of HCV, was also dependent on PLA2G4A. These results highlight previously unrecognized parallels in the assembly pathways of these human pathogens, and define PLA2G4A-dependent lipolysis as crucial prerequisite for production of highly infectious viral progeny.

## Author Summary

The human genome encodes more than 30 phospholipase A2s. These enzymes cleave fatty acids at the C2 atom of phosphoglycerides and thus modulate membrane properties. Among all PLA2s only PLA2G4A, which is recruited to perinuclear membranes by Ca^2+^ and activated by extracellular stimuli via the mitogen activated protein kinase pathway, specifically cleaves lipids with arachidonic acid. Metabolism of arachidonic acid yields prostaglandins and leukotriens, important lipid mediators of inflammation. We show that inhibition of PLA2G4A produces aberrant HCV particles and that infectivity is rescued by addition of arachidonic acid. Our results suggest that a specific lipid (arachidonic acid) is essential for production of highly infectious HCV progeny, likely by creating a membrane environment conducive for efficient incorporation of crucial host and viral factors into the lipid envelope of nascent particles. Strikingly, PLA2G4A is also essential for production of highly infectious Dengue Virus (DENV) particles but not for vesicular stomatitis virus (VSV). These observations argue that HCV and DENV which unlike VSV produce particles at intracellular membranes usurp a common host factor (PLA2G4A) for assembly of highly infectious progeny. These findings open new perspectives for antiviral intervention and highlight thus far unrecognized parallels in the assembly pathway of HCV and DENV.

## Introduction

Approximately 160 million people are chronically infected with hepatitis C virus (HCV) [1]. Without treatment, at least 20% of patients will develop liver cirrhosis and of these, approximately 15% will progress to liver cancer within ten years [2]. HCV is the sole member of the genus Hepacivirus within the family of *Flaviviridae*. Its plus strand RNA genome encodes a polyprotein that is flanked by non-translated regions. Proteolytic processing releases ten viral proteins which coordinate viral RNA replication and particle assembly. The non-structural proteins NS3, NS4A, NS4B, NS5A and NS5B in conjunction with cellular co-factors are both necessary and sufficient to catalyze genome replication [3]. Core protein, envelope protein 1 and 2 (E1, E2) reside in the very N-terminal portion of the viral polyprotein and compose the virus particle encasing the RNA genome. These proteins are essential for virus assembly. Interestingly, the ion channel protein p7, and the NS2 protease also contribute functions to the production of infectious viral progeny [4], [5].

Lipid droplets have been recognized as an essential cellular organelle for production of infectious HCV progeny [6]. During virus production core protein resident on lipid droplets recruits viral proteins and RNA, which is an essential prerequisite for virus production [6]. In turn, core protein is loaded onto these cellular organelles through an interaction with diacylglycerol acyltransferase-1 (DGAT-1) [7], an enzyme which catalyzes the final step in the biosynthesis of triglycerides that is essential for lipid droplet biogenesis [8]. In addition, host factors involved in the biosynthesis and secretion of human lipoproteins have emerged as essential cofactors for virus production. Specifically, apolipoprotein B (ApoB), apolipoprotein E (ApoE) and microsomal triglyceride transfer protein (MTTP) were shown to contribute to virus production [9], [10], [11]. Likely as a consequence, infectious HCV is a “lipo-viro particle” rich in cholesteryl esters and comprising viral proteins, ApoB and ApoE [12], [13].

Cells constantly respond to environmental changes by sensing these alterations through dedicated receptors and associated signaling cascades that reprogram cellular processes. Such signaling-dependent modifications may also influence important cellular HCV dependency factors regulated by these pathways thus providing a lead for identification of novel and possibly druggable host factors crucial for HCV. Using this approach we show that mitogen-activated protein kinase (MAP kinase) regulated enzymatic activity of PLA2G4A is crucial for production of infectious HCV progeny highlighting the intricate involvement of host cell lipids and lipid-modifying enzymes in the replication of this virus.

## Results

### The MAPK/ERK signaling pathway is involved in HCV assembly/release

Cultured cells respond to multiple stimuli by growth factors and hormones present in serum-containing culture media. Therefore, to reduce the complexity of our experimental system we developed a transient virus replication assay and cultured cells in serum-free conditions. When transfecting our infectious firefly luciferase reporter virus genome Luc-Jc1 [14] into highly permissive Huh-7.5 human hepatoma cells [15] cultured in the presence or absence of serum, we measured comparable levels of luciferase activity 4 to 48 h after transfection (Figure S1A). Likewise, transduction of luciferase activity upon inoculation of naïve cells with culture fluid from the transfected cells was similar (Figure S1B). Therefore, in this transient time course viral RNA replication and production of infectious progeny particles was comparable in both serum-free and serum-containing conditions.

**Figure 1:**
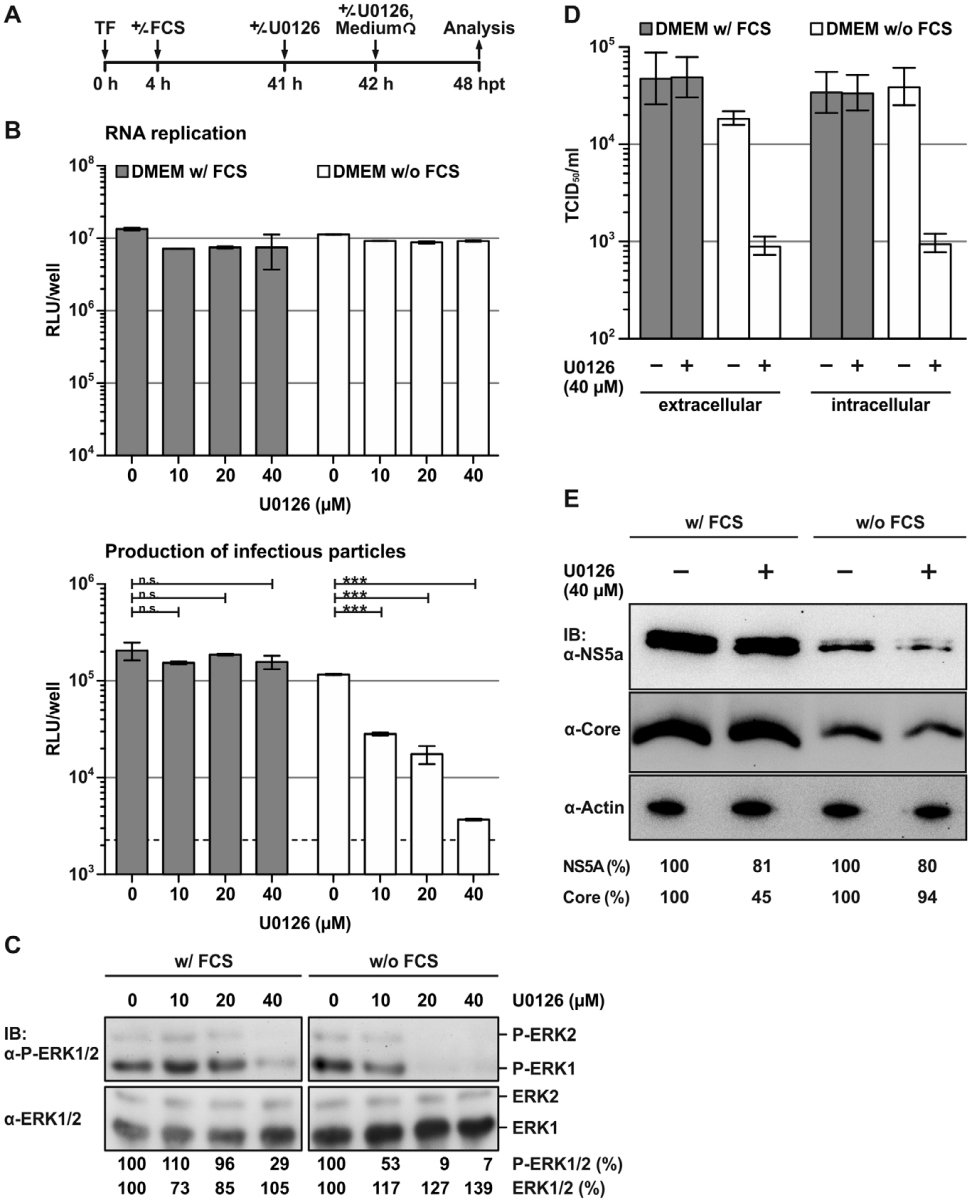
The MAPK/ERK signaling pathway is involved in production of infectious HCV. (A) Schematic representation of the experimental procedure. Huh-7.5 cells were transfected (TF) with Luc-Jc1 RNA [14] and seeded into replicate tissue culture plates. At 4 h post transfection (hpt), medium was changed to culture conditions with or without FCS. Inhibitors (e.g. U0126) were added into the medium at 41 hpt. One hour later, medium was replaced by medium containing given doses of the inhibitor. Finally, at 48 hpt cells and culture fluids were analyzed. (B) HCV RNA replication in cells prepared as in (A) was measured by using a luciferase reporter assays (top panel). The release of infectious particles was determined by inoculation of naïve cells with culture fluids collected at 48 hpt and determination of luciferase activity in cells 72 h after inoculation (bottom panel). Data are shown as means +/− SD of three independent experiments (the dotted line represents background luciferase activity in mock infected cells). (C) Analysis of ERK1/2 expression and phosphorylation in Luc-Jc1 transfected and U0126-treated cells. Cell lysates were collected 48 hpt. ERK proteins were detected using ERK- and phospho-ERK-specific antibodies (bottom and top panel, respectively). (D) Cells were transfected with Jc1 RNA and subjected to the assay described in (A). Culture fluids and cells were harvested 48 hpt and extracellular and intracellular infectivity was determined using a limiting dilution assay. Intracellular infectious particles were collected by three repetitive cycles of freeze and thaw. (E) Aliquots of cell lysates were analyzed for expression of NS5A, core and actin using mono-specific antibodies.

To identify new host factors involved in HCV replication and/or virus production we used pathway-specific inhibitors of central signal transduction cascades including AKT/PKB, mTOR, Wnt, JNK and MAPK/ERK (Figure 1 and data not shown). To reveal rapid, signal-mediated changes of RNA replication and virus production we added the inhibitors 41 h post transfection during the logarithmic growth phase of cells. One hour later culture medium was replaced with fresh medium with or without inhibitors and virus production as well as RNA replication was assessed 6 h later. This procedure which is summarized in Figure 1A ensures that specifically infectivity of particles produced and released during inhibitor treatment – and thus blockade of the respective signaling cascade – is evaluated. Among the inhibitors tested, U0126 a selective inhibitor of the MAPK/ERK pathway, substantially decreased the production of infectious virus particles as is evident from the dose-dependent reduction of luciferase activity in the inoculated cells (Figure 1B). Interestingly, U0126 did not measurably impede RNA replication and only affected HCV particle production when added in serum-free medium. This latter finding may be related to a much more efficient blockade of the MAPK/ERK pathway in serum-free conditions compared to serum-containing medium that is evident from a lower level of phosphorylated ERK1 and ERK2 in the presence of the drug when serum was absent (Figure 1C). Collectively, these results suggested that the applied doses of U0126 efficiently prevented phosphorylation of ERK under serum-free conditions thus impeding production of infectious HCV progeny. Interestingly, PD98059 and Sorafenib which inhibit the MAPK/ERK pathway upstream of U0126 [16] [17] also reduced production of infectious HCV particles (Figure S2) further confirming the role of MAPK/ERK signaling in HCV morphogenesis. Notably, at least under serum free conditions, these inhibitors also reduced RNA replication. This may either be linked to a more potent suppression of MAPK/ERK signaling or due to inhibition of additional signaling events.

To confirm these findings, we analyzed the impact of U0126 on the production of infectious wildtype Jc1 particles [18] in transfected cells (Figure 1D and E). Congruent to our findings with reporter viruses, treatment of cells with U0126 reduced both extracellular and intracellular levels of infectious HCV particles (Figure 1D). Although intracellular levels of core and NS5A were moderately reduced in the absence of serum (Figure 1E), addition of U0126 did not further reduce abundance of viral proteins suggesting that the inhibitor did not prevent RNA translation or RNA replication. We also tested if presence of U0126 interferes with HCV cell entry by adding the drug to infectious reporter virus particles that had been produced in the absence of the inhibitor (Figure S3). Since HCV infection was not decreased, we concluded that addition of U0126 does not prevent HCV cell entry but interferes with production of infectious progeny particles under serum-free conditions.

### The MAPK/ERK regulated PLA2G4A is involved in production of infectious HCV

**Figure 2:**
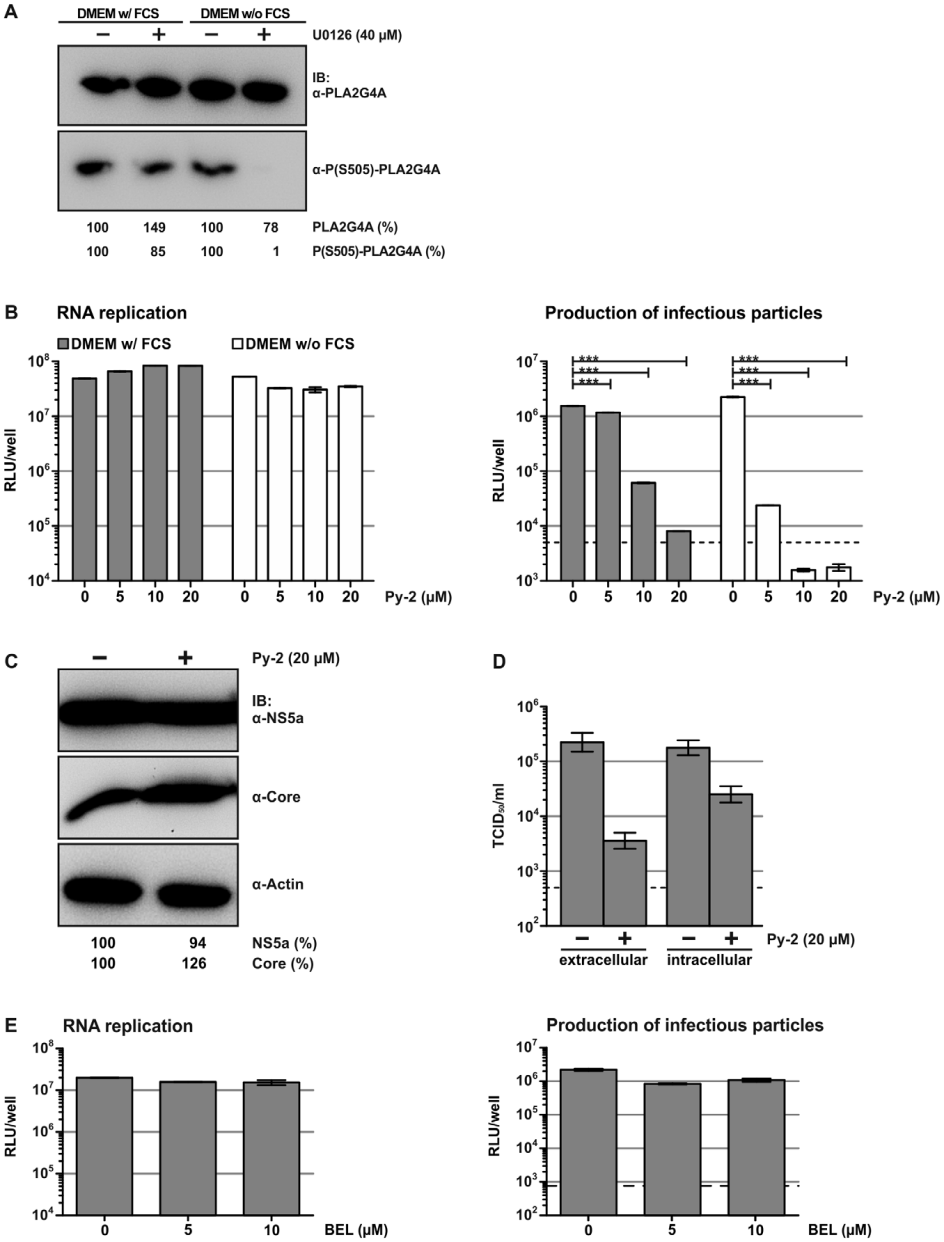
Inhibition of PLA2G4A by Py-2 impairs infectivity of HCV. (A) U0126 prevents phosphorylation of PLA2G4A in the absence of serum. Cells were cultured in presence or absence of serum and the U0126-assay was carried out as described in Figure 1A. Cell lysates were analyzed using antibodies specific for PLA2G4A or the S505-phosphorylated enzyme. (B) Luc-Jc1-transfected cells were treated with indicated doses of Py-2 as outlined in Figure 1A. The influence on RNA replication (left panel) and production of infectious particles (right panel) was determined as described in the legend to Figure 1. Data are shown as means +/− SD of three independent experiments (the dotted line represents background luciferase activity in mock infected cells). (C) Analysis of NS5A and core protein levels in Jc1-transfected cells in the presence or absence of Py-2. (D) Secreted and intracellular infectious HCV was quantified using a limiting dilution assay. (E) An iPLA_2_-specific inhibitor does not impede HCV RNA replication or virus production. Huh-7.5 cells were transfected with Luc-Jc1 and treated with given doses of BEL, an iPLA2-specific inhibitor, using the procedure outlined in Figure 1A. Luciferase activity was measured in transfected (left panel) and in the inoculated cells (right panel). Data are shown as means +/− SD of three independent experiments (the dotted line represents background luciferase activity in mock infected cells).

Besides activating transcription factors in the nucleus, ERK1/2 also directly regulate the activity of cytoplasmic enzymes. Therefore, we searched for cellular factors that are regulated by the MAPK/ERK pathway and operate at the ER, the presumed site of HCV particle production. Based on these criteria we focused on PLA2G4A, which is activated by MAPK/ERK-dependent phosphorylation [19] and recruited to the ER by Ca^2+^ ions [20], [21], as a possible candidate host factor that may be responsible for the observed U0126-dependent blockade of HCV particle production. In line with our finding that U0126 only inhibited HCV in the absence of serum, phosphorylation of PLA2G4A was selectively inhibited under these conditions and not affected when serum was present (Figure 2A).

To investigate if PLA2G4A activity is relevant for the production of infectious HCV particles we treated Luc-Jc1 transfected cells with pyrrolidine-2 (Py-2), a specific inhibitor of this type of phospholipase [22], [23]. Irrespective of culturing these cells in the presence or absence of serum, we observed a strong and dose-dependent inhibition of production of infectious particles resulting in a more than 100-fold reduction of luciferase transduction at 5 and 20 µM of Py-2, respectively (Figure 2B). Similar to U0126, RNA replication and cell entry were not affected by Py-2 (Figure 2B and data not shown). Moreover, accumulation of HCV proteins in cells transfected with authentic HCV was not changed by addition of Py-2 (Figure 2C). However, titers of both extracellular as well as intracellular infectivity were strongly impaired by 50- and 10-fold, respectively (Figure 2D). Interestingly, Py-2 also inhibited production of infectious genotype 1A, 3A and 5A HCVcc particles, indicating that PLA2G4A activity is important for HCV virus production across different HCV genotypes (Figure S4).

Intracellular phospholipase A2 enzymes comprise cytosolic, Ca^2+^-dependent enzymes (cPLA2) as well as the structurally similar Ca^2+^-independent lipases (iPLA2) [24]. To investigate if iPLA2 activity contributes to HCV particle production we treated Luc-Jc1 transfected cells with bromenol lactone (BEL) a specific inhibitor of iPLA2 [25], [26]. However, BEL neither affected HCV RNA replication nor production of infectious particles (Figure 2E), supporting the notion that specifically the PLA2G4A is involved in the HCV life cycle.

### PLA2G4A activity is crucial for high infectivity of Dengue virus (DENV) but not Vesicular stomatitis virus (VSV) particles

**Figure 3:**
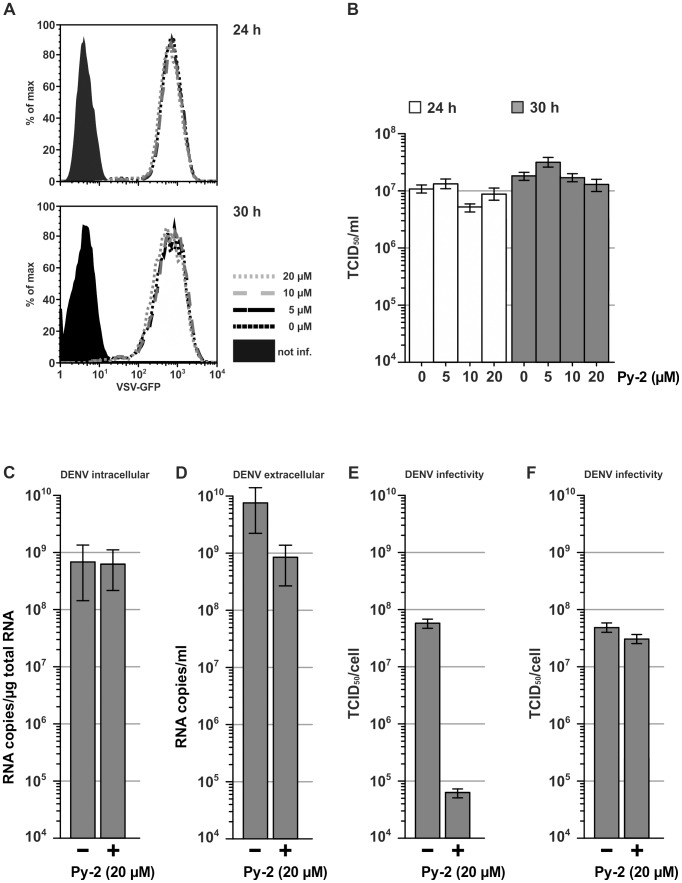
Py-2 inhibits production of infectious particles in a virus-specific fashion. Influence of Py-2 on VSV replication and virus production. (A) Huh7.5 cells were infected with VSV*M_Q_ for 4 h and treated with Py-2 as outlined in Figure 1A. RNA replication and viral protein expression was determined by FACS analysis of GFP expression 24 and 30 h post inoculation. (B) Production of infectious viral progeny at these time points was assessed using a limiting dilution infection assay. The impact of Py-2 treatment on DENV was investigated by inoculation of Huh-7.5 cells with DENV-2 strain 16681 at an MOI of 0.3. The abundance of DENV RNA in the cell lysate (C) and culture fluid (D) at 48 hpi was determined by quantitative RT-PCR (means +/− SD are given). (E) Production of infectious particles was quantified at 48 hpi using a limiting dilution assay. (F) Influence of Py-2 on DENV cell entry was determined by addition of Py-2 to DENV particles prepared in the absence of the drug.

To determine whether utilization of PLA2G4A activity is unique to HCV or common to other enveloped viruses, we analyzed production of infectious VSV and DENV in the presence of Py-2. In case of VSV, a member of the family *Rhabdoviridae* which assembles infectious virus particles at the plasma membrane [27], we used a replication competent reporter virus (designated VSV*M_Q_) that expresses a GFP transgene from an additional transcriptional unit placed between the G and L genes [28]. Interestingly, Py-2 treatment of VSV*M_Q_ infected Huh-7.5 cells did neither affect intracellular level of GFP (Figure 3A) nor production of infectious VSV progeny particles (Figure 3B) indicating that unlike for HCV, production of infectious VSV particles did not rely on PLA2G4A activity. In contrast, production of infectious DENV, a relative of HCV from the genus Flavivirus that is thought to assemble at intracellular membranes [29], was heavily impaired by Py-2 treatment (Figure 3). Strikingly, like for HCV, RNA replication was not affected (Figure 3C) and release of particles was only moderately reduced as is evident from ca. 10-fold lower copy numbers of viral RNA in the culture fluid of Py-2 treated cells compared to mock treated DENV infected cells (Figure 3D). Importantly, when we quantified the infectivity of released particles using a limiting dilution assay we noted an approximately 1,000-folder lower infectivity titer for particles produced in the presence of Py-2 as compared to particles produced in the absence of the compound (Figure 3E). Since Py-2 did not grossly inhibit cell entry (Figure 3F) we concluded that inhibition of PLA2G4A activity via Py-2 primarily impairs infectivity of released particles (Figure 3E). In summary these results indicate that PLA2G4A is a key host enzyme required for efficient release and high infectivity of HCV and DENV, but not VSV particles.

### Knockdown of PLA2G4A increases HCV susceptibility to Py-2

**Figure 4:**
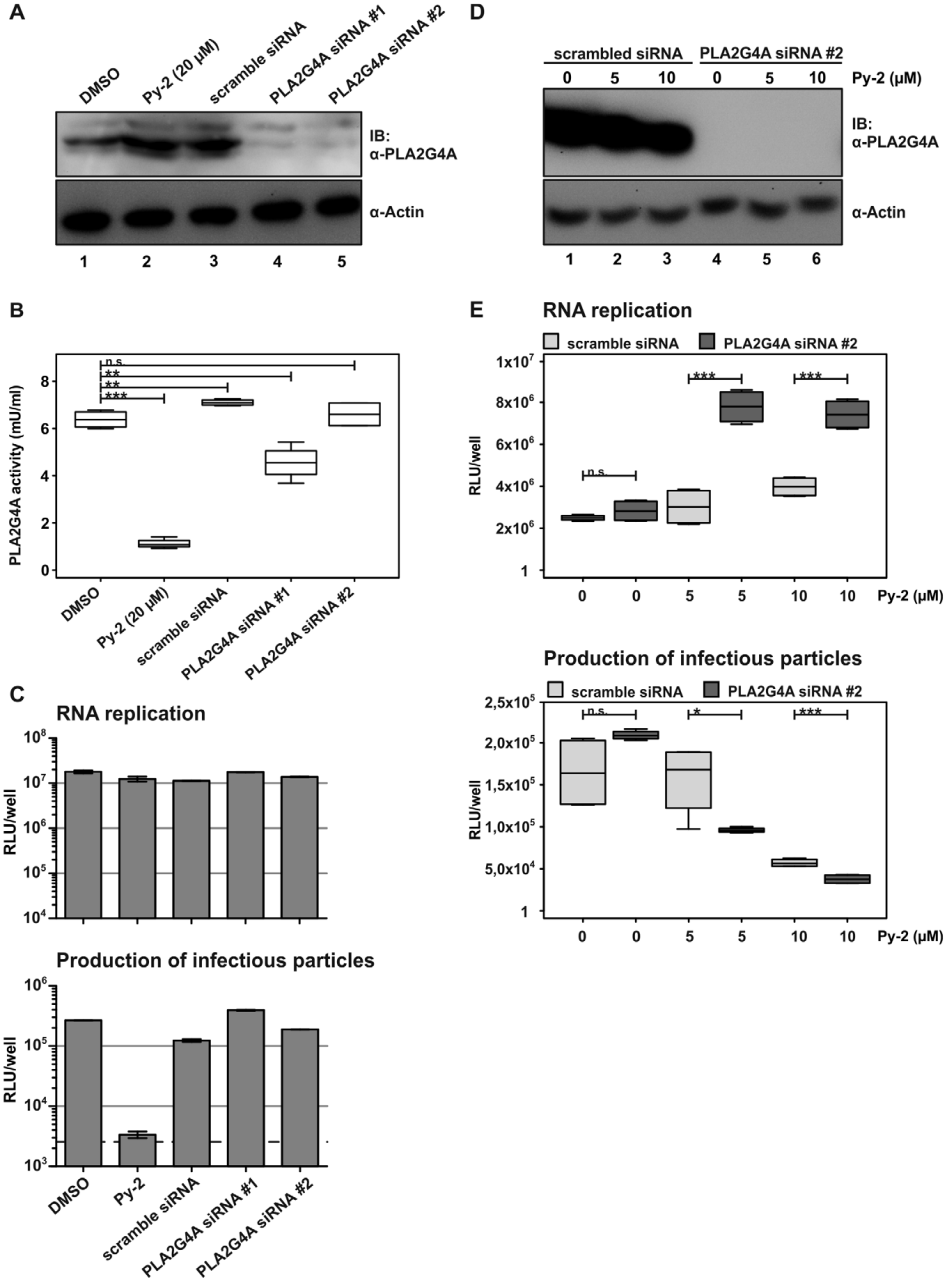
Knock down of PLA2G4A increases susceptibility of HCV to Py-2. Huh-7.5 cells were transfected with PLA2G4A-specific or scrambled siRNAs. 96 h later cells were inoculated with a concentrated Luc-Jc1 virus preparation (A) Efficiency of PLA2G4A knock down was controlled by Western blot. (B) PLA2G4A enzyme activity was measured using a commercial assay. (C) HCV RNA replication was quantified in cell lysates (top panel) and release of infectious particles was determined after inoculation of naïve Huh-7.5 cells (bottom panel). Data are shown as means +/− SD of three independent experiments (the dotted line represents background luciferase activity in mock infected cells). (D and E) Huh-7.5 cells were transfected with PLA2G4A-specific or scrambled siRNAs. 96 h later, cells were infected with Luc-Jc1 particles and subjected to the inhibitor assay outlined in Figure 1A. (D) Knock down of PLA2G4A was monitored by Western blotting. (E) HCV RNA replication was measured in cell lysates (top panel) and release of infectious particles was determined by inoculation of naïve Huh-7.5 cells (bottom panel) and luciferase assays. The box plots in panels (B) and (E) as well as in the following figures visualize the full distribution of the data; the central horizontal line in each box indicates the value of the median, whereas the box represents the range between the lower and upper quartile of the data, i.e. the area in which the central 50% of the measurements lie. The whiskers extend from the quartiles to the minimum and maximum measurements, respectively. Statistical significance of difference between means is indicated using asterisks (*): n.s - not significant, * marginally significant (p≤0.1), ** significant (p≤0.05), *** highly significant (p≤0.01).

To corroborate our finding that PLA2G4A is involved in production of infectious HCV particles we knocked down expression of the enzyme in HCV transfected cells using RNA interference (Figure 4). Surprisingly, despite decreased abundance of PLA2G4A in siRNA-transfected cells (Figure 4A), we observed at best a moderate reduction of the total cellular PLA2G4A activity as determined by a commercial enzymatic test (Figure 4B). In line with the result of the enzymatic test, knock down of PLA2G4A did not impede production of infectious HCV particles (Figure 4C). In contrast, reduction of virus titer correlated again with PLA2G4A inhibition upon treatment with Py-2 (Figures 4B and C).

To confirm that indeed PLA2G4A was directly contributing to HCV particle production rather than alternative enzymes which may share a similar enzymatic activity, we combined siRNA treatment with application of Py-2. Under these conditions the reduction of PLA2G4A within cells should increase the susceptibility of HCV to treatment with Py-2 because due to lower abundance of the host factor a lower level of the drug should be sufficient to prevent HCV particle production. As expected, siRNA and Py-2 treatment did not decrease HCV RNA replication (Figure 4D and E). In fact RNA-replication moderately increased in cells that were treated this way compared to cells receiving a scrambled siRNA and no Py-2 (Figure 4E). Despite of this we found significantly lower levels of infectious virus particles secreted from cells receiving the PLA2G4A-specific siRNA and 5 or 10 µM Py-2 as compared to cells treated with the scrambled siRNA and these drug doses (Figure 4E). Collectively, these data indicate that siRNA treatment does not sufficiently suppress PLA2G4A enzyme activity to limit HCV production in Huh-7.5 cells. However, when adding the PLA2G4A-specific inhibitor to these cells, knock down of PLA2G4A increased the susceptibility to the drug, arguing that the abundance of enzymatically active PLA2G4A is important for production of infectious particles.

### Arachidonic acid (AA) restores efficient production of infectious HCV in the absence of PLA2G4A activity

Mammals encode genes for more than 30 phospholipase A_2_s (PLA_2_-s) and related enzymes which are further divided into several classes [24]. These enzymes share the property of hydrolyzing the sn-2 position of membrane glycerophospholipids to release free fatty acids and lysophospholipids. Among PLA_2_-s only the PLA2G4A displays selectivity for cleaving phospholipids carrying AA at the sn-2 position [30], [31]. While local release of AA modifies membrane properties including curvature and fluidity [32], [33], [34], this lipid is also a precursor for production of bioactive prostaglandins (PGs) and leukotriens (LTs) which play essential roles in inflammatory reactions. In fact, production of PGs and LTs is reduced by ca. 90% in PLA2G4A deficient mice highlighting the dominant role of this phospholipase for generation of these lipid mediators [35], [36]. Given these circumstances we wanted to distinguish if properties of the PLA2G4A-derived cleavage products (AA and/or lysophosphatidic acid) directly contribute to HCV particle production, or if these molecules may indirectly promote virus production through activating inflammatory reactions. Since AA is further metabolized by lipoxygenases and cyclooxygenases to yield prostaglandins and leukotrienes we assessed whether inhibition of AA metabolism by application of (S)-Flurbiprofen or Nordihydroguaiaretic Acid (NDGA), inhibitors of cyclooxgygenases and lipoxygenases, respectively, prevents efficient HCV particle production. However, neither drug modulated HCV RNA replication or decreased production of HCV particles (Figure S5). In fact high doses of (S)-Flurbiprofen even slightly increased levels of infectious HCV (Figure S5A). These results argue against the notion that AA metabolites and their biological activities are crucial for production of infectious HCV.

**Figure 5:**
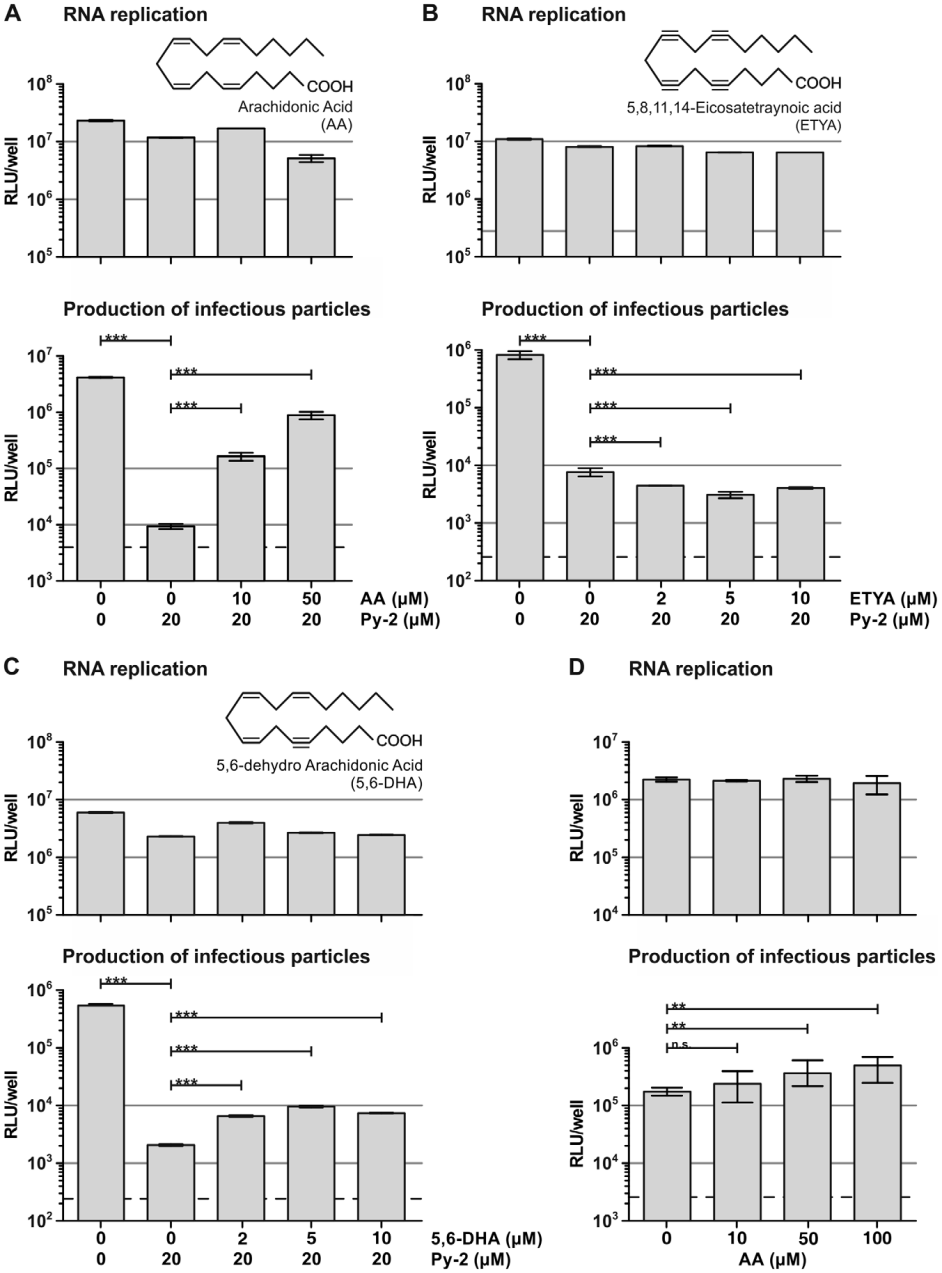
Arachidonic acid restores production of infectious HCV particles in Py-2-treated Huh-7.5 cells. At 32 hpt given doses of (A) arachidonic acid (AA), (B) 5,8,11,14-eicosatetraynoic acid (ETYA) or (C) 5,6-dehydro arachidonic acid (5,6-DHA) were added to the medium of Luc-Jc1 transfected cells. RNA replication and virus production in the presence or absence of Py-2 was determined as described in Figure 1A. Data are shown as means +/− SD of three independent experiments (the dotted line represents background luciferase activity in mock infected cells). (D) RNA replication and virus production in cells treated as above except that medium was only supplemented with AA (and not with Py-2).

Next, we analyzed if application of AA or related fatty acids restores production of HCV particles in the presence of the PLA2G4A inhibitor Py-2. Remarkably, we observed a pronounced and dose-dependent restoration of infectious particle production in the presence of AA (Figure 5A). Importantly, 5,8,11,14-Eicosatetraynoic acid (ETYA) a derivative of AA with four triple bonds at positions 5, 8, 11, and 14 of the acyl backbone did not restore virus production (Figure 5B). Likewise further natural fatty acids with 1, 2, 3 or 4 double bonds at various positions did not relieve the blockade of virus production caused by addition of the PLA2G4A inhibitor (Figure S6). Among all tested lipids only AA itself and to a moderate level 5,6-dehydro AA (5,6-DHA) restored virus production (Figure 5A and C). Notably, AA did not increase HCV cell entry since the infectivity of particles produced in the absence of the drug was not stimulated by addition of AA during cell entry (Figure S7). Next, we investigated if repression of DENV infectious particle production is also relieved by addition of AA. As is depicted in Figure S8, we noted a trend that high doses of AA partially restore production of infectious DENV progeny in the presence of Py-2. However, the rescue of infectious virus production was moderate and not statistically significant.

To investigate if in the context of HCV, addition of AA truly rescues the blockade of PLA2G4A enzymatic activity and not simply over-stimulates production of HCV particles, we applied AA to HCV transfected cells in the absence of Py-2. Interestingly, under these conditions we observed a moderate increase of HCV infectivity (Figure 5D). This finding mirrors the moderate gain of infectivity when cells were treated with Flurbiprofen that prevents AA metabolism. Since both treatments are expected to increase availability of AA, these data suggesting that availability of AA may be sub-saturating in non-treated HCV transfected cells. Collectively, these results indicate that specific properties of AA, which is created by cleavage of glycerophospholipids carrying this lipid at the sn-2 position by PLA2G4A, are important for production of highly infectious HCV progeny particles.

### PLA2G4A contributes to association of core with lipid droplets and to core protein envelopment

Pyrrolidine has been described as precursor for compounds against the NS3-protease and RNA-polymerase of HCV [37]. As the PLA2G4A-specific inhibitor Py-2 shares a heterocyclic ring with pyrrolidine we wanted to exclude that Py-2 may prevent virus production indirectly by inhibiting the viral protease or polymerase. Both enzymes contribute to active RNA replication complexes and may be required to feed in newly synthesized viral RNA into assembling virus particles. To address this, we treated Jc1-transfected cells with a polymerase inhibitor (2′-C-methyladenosine; 2′CMA), a protease inhibitor (boceprevir), with Py-2, or with quinidine, the latter being a class I antiarrhythmic drug recently found to inhibit production of infectious HCV [38]. As expected, only the RNA-replication inhibitors (2′CMA and boceprevir) reduced abundance of HCV RNA in transfected cells (Figure S9A) dose dependently. While at the used doses (well beyond the IC_90_ for all compounds) all drugs moderately impaired release of HCV core protein (2–5-fold), only Py-2 and quinidine strongly reduced infectivity of particles (Figure S9C) to levels more than 20-fold lower compared to the DMSO control. Thus, it is unlikely that indirect effects of Py-2 on RNA-replication are responsible for the reduced amount of secreted particle and their impaired infectivity. Rather these findings argue that Py-2 directly interferes with HCV assembly and the infectivity of released particles.

To investigate how Py-2 impairs HCV assembly, we investigated the subcellular localization of core, and PLA2G4A in the presence or absence of Py-2. Adipose differentiation-related protein (ADRP), a host protein interacting with the surface of LDs was stained in parallel. Since we were unable to detect endogenous PLA2G4A with commercial antibodies, we created a stable Huh-7.5 cell line ectopically expressing a GFP-tagged PLA2G4A protein. As is shown in Figure S10 we did not see gross changes of the distribution of these proteins during the short term Py-2 treatment. Moreover, localization of GFP-PLA2G4A did not differ between cells expressing HCV proteins and those cells that were not positive for HCV. These findings provide preliminary evidence that localization of epitope tagged PLA2G4A is not influenced by HCV. More work, ideally with untagged PLA2G4A will be needed to fully resolve the localization and trafficking of this protein in the presence or absence of HCV and Py-2.

**Figure 6:**
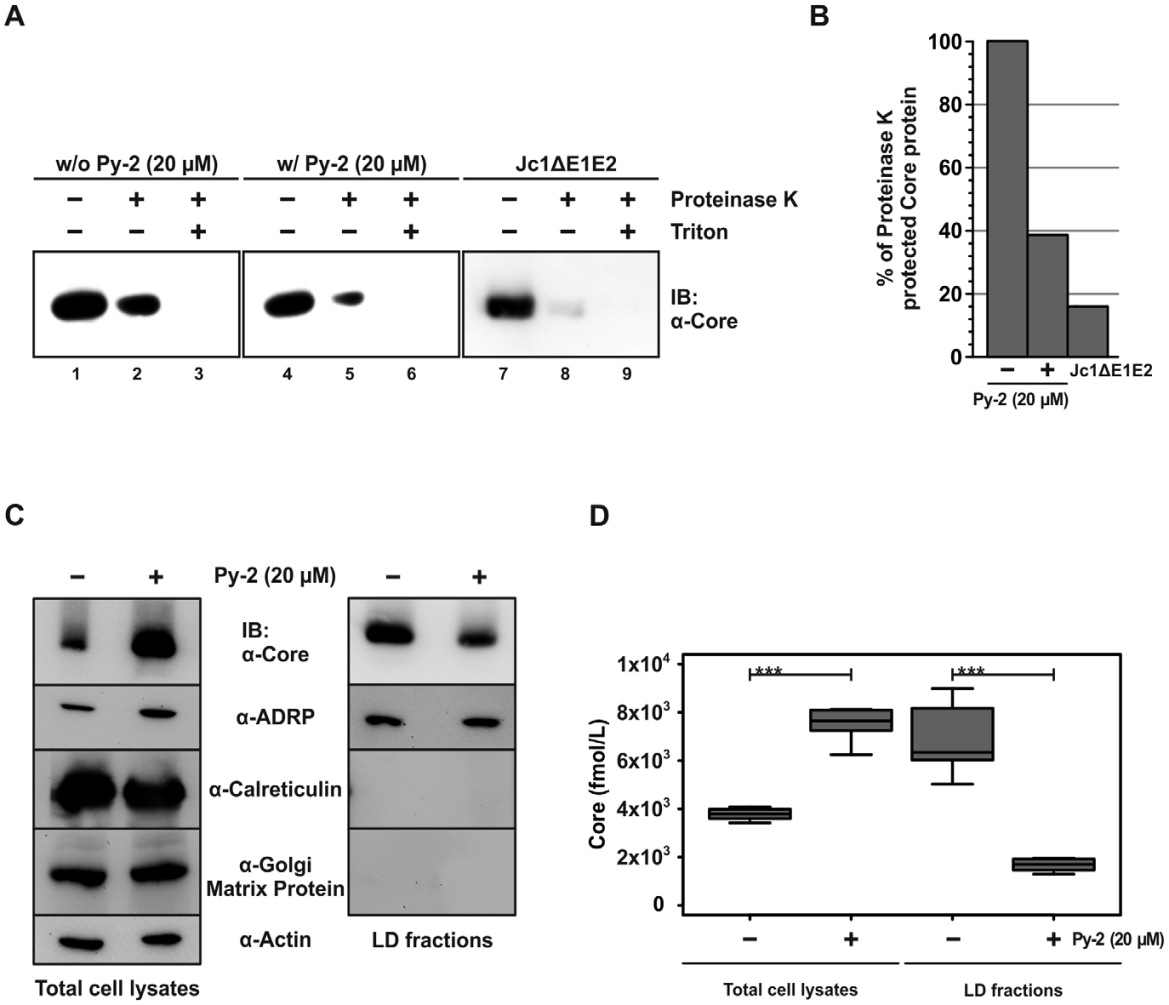
PLA2G4A activity contributes to the association of core with lipid droplets and the membrane envelopment of core. Cells were treated with Py-2 as outlined in Figure 1A. (A) Freeze and thaw lysates of these cells were prepared as described in materials and methods and were left untreated or were incubated with proteinase K in the presence or absence of Triton X-100. The abundance of core protein was determined by Western blot. Cells transfected with a Jc1 mutant lacking the coding region of E1 and E2 served as control. (B) The % of core protein protected from proteolytic digestion was determined by chemiluminescence imaging and evaluated by using the ImageJ software. Mean values of two independent experiments are shown. (C) Total cell lysates were subjected to Western blots for detection of core, ADRP, Calreticulin and Golgi Matrix Protein (left panel). In parallel equal amounts of total cell lysates were used for preparation of lipid droplets by ultracentrifugation. Lipid droplet associated proteins were analyzed by Western blotting (right panel). (D) The amount of core protein in the total lysates and the LD fractions was determined by using a core-specific ELISA. Statistical significance of differences of means: n.s - not significant, * marginally significant (p≤0.1), ** significant (p≤0.05), *** highly significant (p≤0.01).

Next we assessed the amount of intracellular core protein that is resistant to proteolysis by proteinase K. Reasoning that core protein that is surrounded by a membrane should be protected from digestion by the protease, this assay estimates the number of core protein that has acquired a lipid envelope. Since among members of the family flaviviridae virus particle envelopment depends on expression of functional glycoproteins and as for HCV deletion of E1-E2 abrogates production of infectious progeny [39], we used a Jc1 mutant carrying a deletion of E1-E2 genes as a control and reference. As expected, when the protease was added to cell lysates prepared by repetitive cycles of freeze together with detergent (Triton X-100), the viral protein was completely degraded (Figure 6A). However, when cell lysates were incubated with the protease in the absence of detergent, a substantial amount of core protein resisted digestion indicating protection by a membrane envelope. Notably, the amount of protected core protein was approximately 3-fold lower in Py-2 treated compared to DMSO treated Jc1 transfected cells, resembling the phenotype of cells transfected with Jc1ΔE1E2 (Figure 6A and B), arguing that Py-2 had decreased the amount of enveloped core protein structures.

Since trafficking of core protein to lipid droplets (LDs) [6] is crucial for assembly of infectious progeny particles we analyzed the influence of PLA2G4A on the accumulation of core protein on LDs. To this end, we prepared LDs from Jc1-transfected and Py-2 treated cells and analyzed the abundance of core protein on the surface of this cellular organelles. The quality of our LD preparation was monitored by detection of actin (cytosol), ADRP, calreticulin (ER) and Golgi matrix protein (Golgi) in total lysates and the LD fraction (Figure 6C). Importantly, calreticulin and Golgi matrix protein were below the detection limit of our assay in the LD fraction whereas ADRP was readily detected thus confirming that our procedure successfully enriched cellular LDs. Notably, Py-2 moderately increased the total cellular level of core protein but at the same time reduced the abundance of core in the LD fraction of the cell lysate. This difference evident by western blot was further confirmed using a core-specific ELISA demonstrating a ca. 2-fold higher core protein amount in the lysate of Py-2-treated cells and about 3-fold lower levels in the LD fraction. Collectively, these data indicate that Py-2-dependent inhibition of PLA2G4A impedes association of core with LDs which in turn may limit core protein envelopment and particle release.

### Inhibition of PLA2G4A produces HCV particles with aberrant protein composition

**Figure 7:**
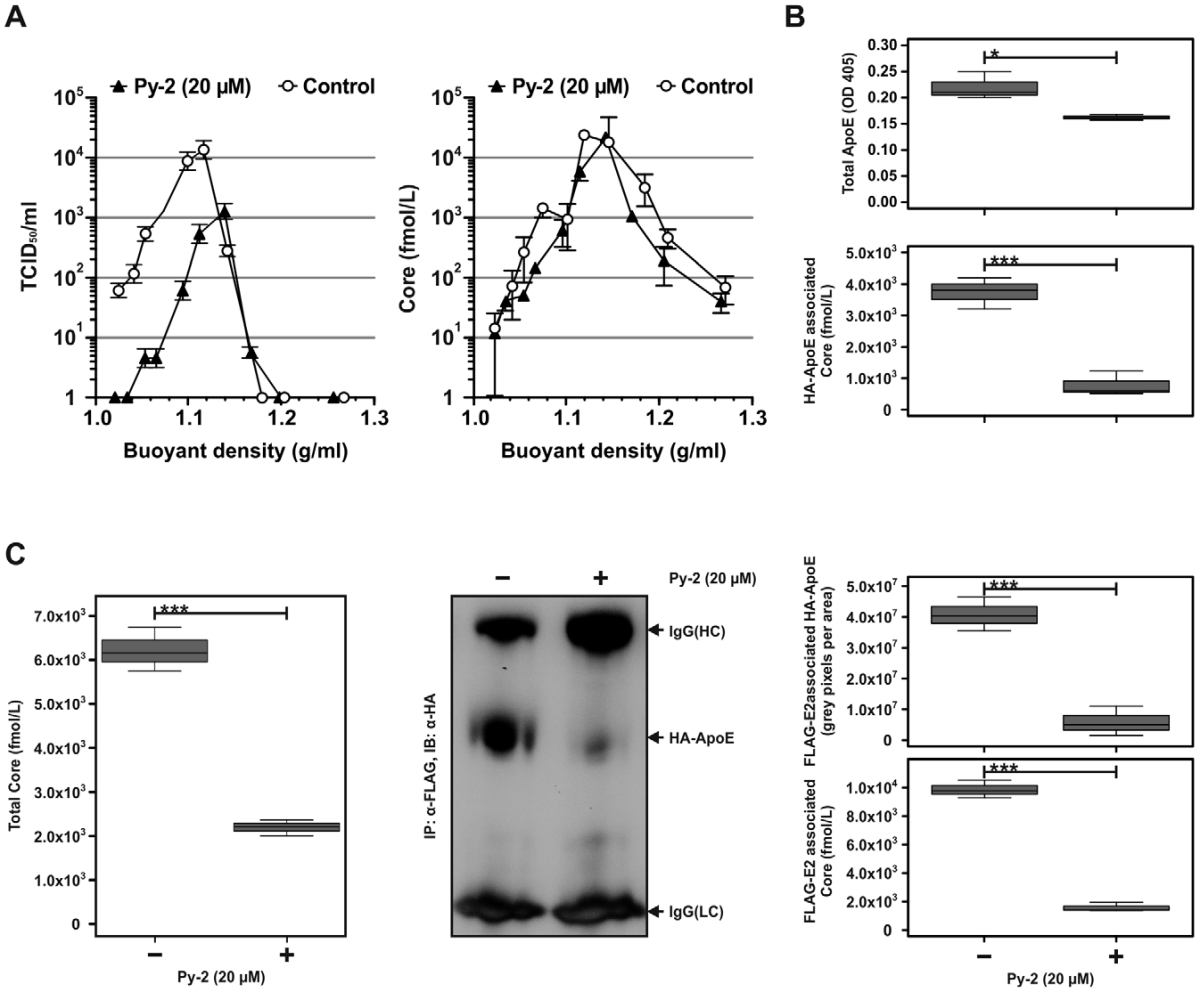
HCV particles produced in the presence of Py-2 display aberrant protein composition. (A) Virus particles produced in the presence or absence of Py-2 were separated by using ultracentrifugation and an iodixanol step gradient. Ten fractions were collected from the bottom, and core protein abundance (left panel) and infectivity titers (right panel) were determined. One example of two independent experiments is given. (B) FLAG-Jc1 RNA [13] was transfected into Huh-7.5-HA-ApoE cells (Figure S11) and cells were treated with Py-2 as outlined in Figure 1A. The total amount of ApoE secreted from transfected cells was determined using an ELISA (top panel). Virus containing culture fluid of Py-2 and untreated cells were normalized for equal quantities of ApoE and precipitated with ApoE-specific antibodies. The amount of co-precipitated core protein was determined by ELISA and is expressed relative to the untreated control. Data are shown as means +/− SD of three independent experiments (C) FLAG-Jc1 transfected Huh-7.5-HA-ApoE cells were treated as in (B). The amount of secreted HCV core protein was determined by ELISA (left panel) and normalized to equal amounts of core prior to precipitation with FLAG-specific antibodies. The amount of co-precipitated ApoE and core was determined by Western blot and ELISA, respectively and is expressed relative to the untreated control. Data are shown as means +/− SD of three independent experiments.

In principle inhibition of PLA2G4A by Py-2 may reduce infectivity by preventing assembly/release of particles and/or by altering particle properties including the association with lipoproteins. Therefore, we used ultracentrifugation of HCV particles through density gradients to analyze the amount and density of virus particles released from cells treated in the presence or absence of Py-2 (Figure 7). Using this approach we noted that the distribution of HCV core protein-containing structures throughout the density gradient was essentially unchanged with peak core protein levels in fractions with a density of 1.11–1.18 g/mL irrespective of Py-2 treatment (Figure 7A). Notably, the overall amount of released core protein was moderately reduced (ca. 2–3-fold; Figure 7A and C) when particles were produced in the presence of the PLA2G4A inhibitor. Most strikingly, inhibitor treatment heavily decreased the infectivity of released HCV particles by 50- to 100-fold (Figure 7A).

To elucidate, why particles produced in the presence of Py-2 are less infectious, we analyzed their protein composition. To this end we transfected Jc1 carrying a FLAG-epitope tag at the N-terminus of E2 [13] into Huh-7.5 cells where endogenous ApoE was silenced by a specific shRNA and replaced by ectopic expression of an HA-tagged, shRNA resistant, human ApoE gene (Figure S11). This approach enabled us to monitor co-precipitation of viral and host factors with an ApoE-specific or a virus envelope-specific antibody. Notably, treatment of transfected cells with Py-2 reduced the amount of secreted ApoE and core protein by ca. 25% and 65%, respectively (Figure 7B and 7C, respectively). Therefore, we normalized the culture fluids to equal quantities of ApoE or core protein before the ApoE-specific or FLAG-specific co-precipitation. Remarkably, Py-2 treatment reduced the level of core protein co-precipitating with ApoE by 5-fold (Figure 7B). Likewise, inhibition of PLA2G4 lowered the association of ApoE and core with the FLAG-tagged E2 by ca. 5-fold (Figure 7C). In summary, inhibition of MAPK-dependent PLA2G4A activity by Py-2 moderately decreased the titer of released HCV particles but heavily impaired infectivity of both intracellular and extracellular particles likely through gross changes of their protein composition.

## Discussion

In this study we manipulated key cellular signaling cascades to identify host cellular HCV dependency factors. We report that blockade of the MAPK/ERK cascade by a well-established specific inhibitor (U0126) potently repressed production of infectious HCV progeny (Figure 1). Making reasonable assumptions (activation by ERK, function at the ER membrane), we focused on PLA2G4A, an ERK-regulated host enzyme that is recruited to the ER-membrane by Ca^2+^, as possible new HCV dependency factor for HCV assembly. Our further experiments provide three lines of evidence supporting our conclusion that the ERK-dependent activation of PLA2G4A is crucial for production of infectious progeny:

First, we show that Py-2 impedes production of infectious HCV in a dose-dependent fashion (Figure 2). Notably, phospholipase A_2_ enzymes are subdivided into several classes including secreted PLA_2_s (sPLA_2_s), Ca^2+^-dependent cytosolic (cPLA_2_s), CA^2+^-independent cytosolic (iPLA_2_s), platelet-activating factor acetylhydrolases (PAF-AHs), lysosomal PLA_2_s and the most recently identified adipose-specific PLA [24]. Importantly, Py-2 potently inhibits PLA2G4A (the α isoform of the Ca^2+^-dependent cytosolic PLA_2_s, also named cPLA_2_α) in various in vitro assays [22]. In contrast it interferes with PLA2G4B (cPLA_2_β) and PLA2G4C (cPLA_2_γ) only at very high doses, probably by a non-specific mechanism, and it does not inhibit the secreted sPLA_2_s [22]. Congruently, BEL, a “suicide substrate” and specific inhibitor of iPLA_2_s with a >1,000-fold selectivity for iPLA_2_s over cPLA_2_s [26], did not impede HCV particle production (Figure 2).

Second, using RNA interference we show that reduction of the abundance of PLA2G4A enzyme increased susceptibility of HCV to inhibition by Py-2 (Figure 4). Surprisingly, we did not observe an influence of the knock down of PLA2G4A on HCV particle production (Figure 4). However, we note that in spite of clearly reduced abundance of the enzyme in siRNA-treated cells we measured only a small decline of PLA2G4A enzyme activity. It is possible that the active, phosphorylated PLA2G4A enzyme has a relatively long half-life which may preclude reduction of the active enzyme to a level that limits HCV infectious particle production under these experimental conditions.

Third, we observed an almost complete restoration of HCV infectivity upon supplementing Py-2 treated cells with AA (Figure 5). Importantly, among all PLA_2_ enzymes, only the PLA2G4A displays a preference for cleaving glycerophospholipids carrying the polyunsaturated AA at the sn2-position [24]. Moreover, related fatty acids including ETYA which differs from AA only by triple-bonded C-atoms in place of the double-bonded C-atoms in AA, were unable to restore virus production (Figure 5 and S7). Even 5′6-DHA (with a single triple-bonded C atom) only partially compensated production of infectious HCV in the presence of Py-2 indicating that highly specific properties of AA, the cleavage product of PLA2G4A, are crucial for production of infectious HCV progeny. AA is the precursor for biologically active lipid mediators including prostaglandins, thromboxane and leukotrienes collectively termed eicosanoids. These molecules are synthesized from AA through the cyclooxygenase and lipoxygenase pathways and play important roles during inflammation. However, since inhibitors of both pathways of AA metabolism did not impede production of infectious HCV particles (Figure S6), we believe that the properties of AA itself rather than indirect effects caused by AA-metabolites are important for HCV.

Our data support the conclusion that PLA2G4A activity is relevant for HCV assembly in two principal ways. First, inhibition of PLA2G4A decreased the amount of core protein associated with lipid droplets and reduced the level of core protein that is protected from proteolytic digestion (Figure 6). The latter finding may indicate a lower level of intracellular core protein that is encased in membranes – possibly within virus particles – and therefore protected from proteolysis. Moreover, we observed reduced levels of extracellular HCV particles (2–3-fold; Figure 7). It is currently unclear why inhibition of PLA2G4A reduces the level of core protein at LDs. In principal several mechanisms account for this including an increase of core assembly and subsequent unloading from LDs or a reduced trafficking of core to LDs possibly due to aberrant processing of the protein by signal peptide peptidase cleavage. However, since we observed lower levels of secreted virus particles we consider it unlikely that increased assembly and protein unloading from LDs is responsible. Moreover we did not detect an overt processing defect of core in the presence of Py-2 (Figure 2 and 6). Notably, Gubern et al. have shown that MAPK-dependent phosphorylation of PLA2G4A at Ser^505^ is necessary for biogenesis of lipid droplets [40], [41]. Therefore, it is tempting to speculate that blockade of PLA2G4A by Py-2 reduces lipid droplet biogenesis, thus limiting abundance of core protein at these organelles which are essential for HCV particle production [6]. Assuming that core protein has to be unloaded from LDs to drive budding and virus production which is consistent with the recent findings of Counihan et al. [42], it is reasonable to suggest that reduced core protein levels at LDs may decrease membrane envelopment and particle release. While AA (the product of the PLA2G4A-catalzed triglyceride cleavage) is apparently not required for lipid droplet biogenesis [40], it nevertheless seems to be essential for the second prominent influence of PLA2G4A on HCV, i.e. the production of highly infectious HCV progeny. Remarkably, both HCV and DENV produced and released in the presence of Py-2 were approximately 100-fold less infectious as compared to viruses assembled in the absence of the drug (Figures 2 and 3). Since addition of Py-2 to particles generated in the absence of the drug, did not impede cell entry (data not shown and Figure 3), we exclude that Py-2 interferes with cell entry of HCV or DENV. Rather, our results indicate that particle properties are altered when Py-2 is present. While the density spectrum of released HCV particles was unchanged, co-precipitation with ApoE- or envelope-specific antibodies provide firm evidence that blockade of PLA2G4A disturbs the composition/structure of released HCV particles. Specifically, we noted 5-fold reduced levels of core co-precipitating with anti-ApoE and likewise 5-fold lower amounts of ApoE and core co-precipitating with the envelope protein-specific pull down. These results argue that either less particles are directly associated with ApoE or that particles contain lower levels of ApoE. Since ApoE is important for infectivity of HCV particles [43], [44], a defect in the loading of this protein onto HCV particles may explain their reduced infectivity. In the envelope-specific pull down mediated by the FLAG-tagged viral E2 protein, we observed both 5-fold lower ApoE and also core protein. On one hand this may indicate lower abundance of both proteins in secreted enveloped HCV particles. On the other hand, this may reflect incorporation of lower numbers of glycoprotein complexes into the viral envelope and in turn reduced precipitation efficiency. Unfortunately, due to insufficient sensitivity of currently available envelope protein detection systems, we are currently unable to distinguish between these two possibilities. Nevertheless, these results argue that blockade of PLA2G4A by Py-2 prevents normal loading of viral (core and envelope proteins E1 E2) and host proteins (ApoE) onto nascent HCV particles. As a consequence, virus attachment or the interaction with entry factors or membrane fusion might be inefficient. Notably, recent evidence suggests that AA and other poly-unsaturated fatty acids increase membrane fluidity [32], [33], [34]. Thus, PLA2G4A activity in the vicinity of particle production may modify membrane characteristics including curvature and fluidity. These altered properties may disturb virus budding and/or the trafficking of viral and host-derived components to the site of virus assembly and thus result in the production of particles with disturbed stoichiometry, aberrant envelope composition, and poor infectivity. A more detailed proteomic and lipidomic comparison between particles produced in the presence or absence of Py-2 should help to clarify this in the future.

Our finding that HCV and DENV particle assembly depend on PLA2G4A activity while VSV apparently does not rely on this host factor indicates fundamental differences between the assembly of enveloped VSV particles compared to HCV and DENV. It remains to be shown how exactly PLA2G4A contributes to production and release of infectious DENV particles. While it has been reported that DENV may also usurp LDs for its assembly [45], Welsch and colleagues showed that DENV assembly sites are physically linked to RNA replication sites on modified ER structures [29]. Unlike for HCV, AA did not consistently restore virus production of DENV supporting the notion that PLA2G4A may participate in HCV and DENV morphogenesis through different mechanisms. More work will be needed to fully understand the role of this host factor for these two viruses. Along these lines it will be interesting to analyze if other enveloped viruses (e.g. Coronaviruses) that like HCV and DENV assemble progeny particles at intracellular membranes depend on PLA2G4A as well. Such studies could in the future reveal common replication mechanisms between HCV and DENV (and possibly other viruses) that may provide valuable insights into conserved assembly pathways of enveloped viruses. Finally, inhibitors of the PLA2G4A which have been pursued and brought into clinical development for treating inflammatory diseases may prove useful as antiviral therapeutics for the treatment of chronic HCV infection and possibly other viral diseases.

## Materials and Methods

### Reagents and antibodies

Antibodies were obtained from the following companies: Apolipoprotein B (*Millipore*); PLA2G4A (*Abcam*); P(S505)- PLA2G4A, ERK1/2, P-ERK1/2 (*Cell Signaling*); Actin (*Sigma-Aldrich*); ADRP (*Probiogen*); Golgi Matrix Protein (*Epitomics*); Calreticulin (*Stressgen*); HA-Epitope (*Covance*). Anti-NS5A antibodies (9E10 mouse monoclonal antibody) were kindly provided by C. Rice (Rockefeller University, New York), anti-Core (C7-50 mouse monoclonal antibody) was a kind gift of D. Moradpour (Centre Hospitalier Universitaire Vaudois, Lausanne), and sheep polyclonal antibodies against ADRP were kindly provided by J. McLauchlan (MRC Virology Unit, Institute of Virology, Glasgow, UK). Secondary antibodies were ordered from *Life Technologies*. 2′CMA was kindly provided by T. Tellinghuisen (Department of Infectology, The Scripps Research Institute, Florida). Reagents were ordered from various manufacturers: Pyrrolidine-2 (CAS Registry No.: 337307-06-9; Order ID: 525143, *Merck Chemicals*; U0126, PD98059 (*Cell Signaling*); Sorafenib (BAY 43-9006, *Alexa Biochemicals*); Proteinase K (*Roche*); cPLA2α-siRNA (*Thermo*); ETYA; 5,6-dehydro arachidonic acid; (S)-Bromoenol lactone; NDGA (*Cayman*); Quinidine; arachidonic acid (*Sigma*); FLAG-agarose (*Sigma*).

### Cell culture

Huh-7.5 cells were grown in *Dulbecco's modified minimal essential medium* (DMEM; *Life Technologies*) supplemented with 2 mM L-glutamine, nonessential amino acids, 100 U/ml of penicillin, 100 µg/ml of streptomycin, and 10% fetal calf serum (DMEM w/FCS).

### Constructs

The plasmids pFK-Luc-Jc1 and pFK-Jc1, encoding the genotype 2a/2a chimera Jc1 with or without the firefly luciferase reporter gene have been described [14], [18]. Chimeric HCV constructs pFK-H77/C3 encoding the genotype 1a/2a chimera [18], pS52/JFH1 (A4550C) encoding the genotype 3a/2a chimera [46], and pSA13/JFH1 (C3405G-A3696G) encoding the genotype 5a/2a chimera have been described [47]. For the shRNA-mediated knockdown of ApoE expression, the vector pLenti-3′-U6-EC-EP7 [48] which contains a blasticidine resistance gene was modified to harbor an shRNA specific to the 3′-untranslated region of human ApoE (5′-GCCGAAGCCTGCAGCCATGCG-3′). As control, a construct containing a non-targeting shRNA was used. The lentiviral plasmid pWPI hApoE-Linker-HA-Gun encodes the human ApoE3 variant with an HA tag added to the 3′-end via a Glycine-Glycine-Serine-Glycine linker in the self-inactivating pWPI vector [49] which comprises a GFP-ubiquitin-neomycinphosphotransferase fusion protein as selectable marker. The gene encoding PLA2G4A (*Thermo Scientific*, cDNA clone MHS4426) was N-terminally fused to a GFP-tag and subcloned into the pWPI vector. The creation of a Huh-7.5-cell line expressing GFP-PLA2G4A fusion protein is described below. Finally, pFK-Jc1-ΔE1-E2 was created by a PCR-based strategy. In this construct the entire E1 and E2 coding region is deleted and the core coding region is directly fused in frame to the coding region of p7. Sequence information is available upon request.

### Virus production and inhibitor assays

HCVcc particles and firefly luciferase HCV reporter virus were generated as reported previously [50]. Luciferase reporter virus infection assays were carried out as described [50]. For inhibitor assays, cells were pre-treated with cellular or viral inhibitors to completely abolish enzyme activity 41 h after transfection of HCV-RNA. One hour later, supernatants were removed and fresh medium with inhibitors was added to harvest newly synthesized virus in a period of six hours. At 48 hpt, supernatants and cell lysates were used for the infection of naïve Huh-7.5 cells or subjected to assays as described below.

### Isolation of lipid droplets

Lipid droplets were isolated according to a published protocol [51] with minor modifications. Briefly, Jc1-transfected cells of 10×100-mm plates were scraped into 50 ml PBS and pelleted by centrifugation at 1000×*g*. Cells were resuspended in ice-cold HLM buffer (20 mM Tris-HCl (pH 7.4); 1 mM EDTA) with protease inhibitors (complete Mini; *Roche*) and incubated for 10 min on ice. Cells were homogenized by eight gentle strokes with a Potter-Elvehjem tissue homogenizer and nuclei were removed from lysates by low-speed centrifugation (1000×*g*). Density of the post-nuclear supernatant was adjusted with sucrose (20% final) and samples were loaded below a discontinuous sucrose gradient (0, 5, 20%). Flotation of lipid droplets through the gradient was achieved by centrifugation at 28,000×*g* for 30 min, 4°C. The white band containing lipid droplets at the top of gradient was collected and proteins were characterized by immunoblotting or core ELISA.

### Proteinase K protection assay

Confluent Jc1- transfected cells were suspended in 170 µl PK buffer (50 mM Tris-HCl (pH 8.0); 10 mM CaCl_2_; 1 mM DTT) and homogenized by five freeze-and-thaw cycles. The lysate was divided into three 50 µl fractions and treated with or without 50 µg/ml proteinase K for one hour on ice. As control, the third sample additionally containted 5% (v/v) triton X-100 during protease digestion. The reactions were stopped by 5 mM PMSF for 10 min on ice and addition of Laemmli buffer. Samples were analysed by immunoblotting.

### RNA interference

Huh-7.5 cells were transfected with siRNAs specific to PLA2G4A (D-009886-01, -02; *Thermo*) or scramble siRNA (4390846; *Ambion*) in a forward transfection procedure according to the manufacture's protocol (RNAiMax; *Life Technologies*). To achieve an efficient knock-down, cells were transfected twice within 96 h and re-seeded in 6-wells at a density of 2.5×10^5^ cells per well. Cells were infected with a 20-fold concentrated stock of Luc-Jc1 virus and 41 h later the inhibitor assay was performed as described above. The efficiency of PLA2G4A knock-down was verified by immunoblotting.

### Quantification of HCV RNA and Core protein

Viral RNA was prepared from infected cells using a Nucleo Spin RNAII kit (*Macherey-Nagel*) according to the manual's instructions. 5 µL of the RNA sample was used for HCV-specific quantitative reverse transcription-PCR (qRT-PCR) analysis using a LightCycler 480 device (*Roche*).

HCV-specific qRT-PCRs were conducted in duplicate measurements as published [52] utilizing a one-step RT-PCR LightCycler 480 RNA Master Hydrolysis Probes kit (*Roche*) and the following HCV-specific probe (*Molecular Biosystems*) and primers (*MWG-Biotech*): HCVMGB2 [5′-6FAM (carboxy fluoresceine)-CACGGCTAGCTGTG-MGB-3′]; XTF5 (5′-GTGGCTCCATCTTAGCCCTAGT-3′); and HCMgR2 (5′-TGCGGCTCACGGACCTTT-3′).

To normalize for equal quantities of total RNA in the samples, the GAPDH-specific mRNA was detected in parallel employing GAPDH-specific oligonucleotides (S-GAPDH, 5′-GAAGGTGAAGGTCGGAGTC-3′; A-GAPDH, 5′-GAAGATGGTGATGGG ATTTC-3′) and a GAPDH-specific probe (*TIB Molbiol*), 640-GAPDH-BBQ probe (5′-LC640-CAAGCTTCCCGTTCTCAGCCT-BBQ-3′). Reactions were performed in three stages by using the following conditions: stage 1 (RT), 3 min at 63°C; stage 2 (initial denaturation), 30 s at 95°C; stage 3 (amplification), 45 cycles of 10 s at 94°C and 20 s at 58°C. The amount of HCV RNA was calculated by comparison to serially diluted in vitro transcripts and normalized to the amount of GAPDH, which served as a housekeeping gene. HCV Core protein within cell lysates and culture fluids was quantified with a commercially available diagnostic kit (Architect Anti-HCV; *Abbott*).

### Quantification of DENV RNA

One µg of total RNA or 1/5 of RNA extracted from 100 µl cell culture supernatant was reverse transcribed into cDNA using the High Capacity cDNA Reverse Transcription Kit (*Applied Biosystems*) following the manufacturer's protocol. Quantitative RT-PCR was performed on an ABI PRISM 7000 Sequence Detection System (*Applied Biosystems*). The reaction was carried out in a final volume of 15 µl, including 7.5 µl 2× Green DYE master mix (P.J.K., Kleinblittersdorf), 2 µl cDNA template, 1.5 µl primer mix (5 µM each), 4 µl RNase-free sterile water. Reactions were carried out using the following settings: 95°C: 10 min→40× [95°C: 30 sec→55°C: 60 sec→72°C: 60 sec].

The amounts of DENV RNA were calculated from a standard curve derived from serially diluted *in vitro* transcripts of known concentration. Primers used for the amplification were: sDV2-9687, 5′-GCCCTTCTGTTCACACCATT-3′ and asDV2-9855, 5′-CCACATTTGGGCGTAAGACT-3′.

### Quantification of core and apolipoprotein E

To quantify core protein, cell culture supernatants or immunoprecipitated FLAG-Jc1 particles were diluted in PBS/1% Triton in a ratio of 1∶30. The core ELISA was performed with a commercially available diagnostic kit (Architect Anti-HCV, *Abbott*). ApoE was quantified according to the manufacturer's instructions (*MabTech*).

### Analysis of HCV particles by density gradients

Density gradient centrifugation was performed as described recently [53]. Briefly, viruses were separated by overnight centrifugation through a 0% to 40% iodixanol step gradient at 154,000×*g*. Ten fractions of 1 ml were collected from the bottom and analyzed for virus infectivity, core protein levels, and viral RNA copies.

### Co-immunoprecipitation analysis

Following Py-2 treatment, aliquots of cell culture supernatants were subjected to core or ApoE ELISA in order to equilibrate the volumes for immunoprecipitations. To capture FLAG-Jc1 particles or HA-ApoE, equilibrated supernatants were mixed with either 20 µl FLAG-agarose or 3 µg anti-HA antibody and 30 µl G-protein agarose (*Roche*) overnight at 4°C in gentle rotation. Immunoprecipitated proteins were washed three times in PBS, eluted with 50 µl PBS/1% Triton for 10 min at 50°C and analyzed by core ELISA or immunoblotting.

### Determination of PLA2G4A enzyme activity

Phospholipase A2 activity was measured according to the manufacture's protocol (EnzChek Phospholipase A2 kit; *Life Technologies*). In brief, Huh-7.5 cells were harvested from 35-mm wells, resuspended in 200 µl *EnzChek PLA2 reaction buffer* and disrupted by sonication. To avoid any measurement of iPLA2 activity, Bromenol lactone was added to all samples at a concentration of 5 µM. Liposomes were prepared with the *EnzChek Phospholipase A2 substrate* and mixed with lysates at a ratio of 1∶1 to give a total volume of 100 µl. Samples were transferred in 96-wells and PLA2G4A activity was monitored by the intensity increase of a single wavelength at 515 nm in a fluorescence microplate reader (FLx800; *BioTek*).

### Construction of stable cell lines

For the generation of stable Huh-7.5-HA-ApoE cells, lentiviral gene transfer was used as described before [54]. Endogenous ApoE expression in Huh-7.5 cells was silenced using pLenti-3′-U6-EC-EP7 [48] which contains a blasticidine resistance gene and an shRNA specific to the 3′-untranslated region of human ApoE (5′-GCCGAAGCCTGCAGCCATGCG-3′). Subsequently, ApoE expression was restored by transduction with pWPI hApoE-Linker-HA-Gun described above. Lentiviral particles were generated by transfection of pCMV ΔR.74, pcz VSV-G and a derivative of either pLenti-3′-U6-EC-EP7 or pWPI at a ratio of 3∶1∶3 into 293T cells. Lentiviral particles were collected 48 h post transfection and used to transduce target cells. Selection was carried out in the presence of 5 µg/ml Blasticidine or 0.75 mg/ml G418. For generation of Huh-7.5-GFP-PLA2GA4 cells, Huh-7.5 cells were transduced with lentiviruses carrying the pWPI-GFP_PLA2G4A vector. Transduced cells were selected in the presence of 5 µg/mL Blasticidine.

### Fluorescence microscopy

The protocol for immunostaining was carried out as described previously (Frenzen, Hueging, Steinmann, PLoS Pathogens April 2011 Volume 7 Issue 4 e1002029). Immunostainings of Core and ADRP proteins were performed at dilutions of 1∶7000 respectively 1∶500. Texas Red and Cy-5 secondary antibodies were used at dilutions of 1∶1000.

### Statistical analysis

Statistical data analysis was performed using the free statistical environment R. Data were initially visualized using histograms, boxplots and QQ-plots, and normality of the distributions was assessed. Statistical significance of differences was then calculated using Welch's t-test if data were sufficiently well approximated by a normal distribution, or using the Wilcoxon rank sum test as a non-parametric alternative for non-normal data. P-values were calculated and statistical significance reported as highly significant (***) if p≤0.01, significant (**) if p≤0.05, and marginally significant (*) if p≤0.1. Differences were considered not significant (n.s.) for p>0.1.

## Supporting Information

Figure S1 **HCV RNA replication and virus production in Huh-7.5 cells cultured in the presence or absence of FCS.** (A) Cells were transfected with Luc-Jc1. Four hours later medium was changed to culture fluid with or without 10% FCS. Cells and culture fluids were collected at given time points and RNA replication was determined by luciferase assays. (B) For detection of infectious particles, naïve Huh-7.5 cells were inoculated with the cell free supernatants harvested at the indicated time points. Luciferase activity in the inoculated cells was measured 72 h later. Data are shown as means +/− SD of three independent experiments. The dotted line represents background luciferase activity in mock infected cells.
(TIF)

Figure S2 **The MAPK/ERK inhibitors Sorafenib and PD98059 inhibit production of infectious HCV.** (A, B) Cells were treated with the inhibitors as outlined in Figure 1A. HCV RNA replication in cells was measured by using a luciferase reporter assays (top panels). The release of infectious particles was determined by inoculation of naïve cells with culture fluids collected at 48 hpt and determination of luciferase activity in cells 72 h after inoculation (middle panels). Data are shown as means +/− SD of three independent experiments (the dotted line represents background luciferase activity in mock infected cells). The bottom panels display ERK1/2 expression and phosphorylation in Luc-Jc1 transfected and inhibitor treated cells. ERK proteins were detected as described in Figure 1.
(TIF)

Figure S3 **Influence of MAPK/ERK inhibitor U0126 on HCV cell entry.** Luc-Jc1 particles prepared in the presence or absence of FCS were supplemented with the given dose of U0126 or left untreated. Virus suspensions were incubated with Huh-7.5 cells for 4 h at 37°C. Subsequently, unbound particles as well as the inhibitors were removed and cells were cultured in FCS-containing culture fluid until the analysis of HCV infection 72 h later. Data are shown as means +/− SD of three independent experiments.
(TIF)

Figure S4 **Py-2 impedes production of infectious HCV across different HCV genotypes.** Cells were transfected with indicated chimeric HCV genomes encoding structural proteins of genotype 1a, 3a or 5a, and subsequently treated with Py-2 as described in Figure 1A. Production of infectious progeny was quantified using a limiting dilution assay. Two independent experiments are shown in the two panels. Mean values of six replicates +/− SD of the replicates are given.
(TIF)

Figure S5 **Blockade of arachidonic acid metabolism by inhibition of cyclooxygenases and lipoxygenases does not impede production of infectious HCV.** Luc-Jc1 transfected Huh-7.5 cells were treated with given doses of (S)-Flurbiprofen (A) or NDGA (B) as outlined in Figure 1A. RNA replication in transfected cells and release of infectious particles was determined by luciferase asssays. Data are shown as means +/− SD of three independent experiments (the dotted line represents background luciferase activity in mock infected cells).
(TIF)

Figure S6 **Fatty acids with varying degree of unsaturation are unable to restore virus production in Py-2-treated Huh-7.5 cells.** Luc-Jc1-transfected cells were loaded with given lipids 32 hpt and subsequently subjected to the Py-2 inhibition assay outlined in Figure 1A. HCV RNA replication and virus production was determined by luciferase assays in cells treated with different fatty acids Data are shown as means +/− SD of three independent experiments (the dotted line represents background luciferase activity in mock infected cells).
(TIF)

Figure S7 **Arachidonic acid does not increase HCV cell entry.** Luc-Jc1 particles were supplemented with AA or left untreated. Virus suspensions were incubated with Huh-7.5 cells for 4 h at 37°C. Subsequently, unbound particles as well as the inhibitors were removed and cells were cultured in FCS-containing culture fluid until the analysis of HCV infection 72 h later. Data are shown as means +/− SD of three independent experiments.
(TIF)

Figure S8 **Influence of AA production of infectious DENV particles in the presence or absence of Py-2.** Cells were transfected with a DENV RNA and treated as described in Figure 1A. Infectivity of released particles was determined by inoculation of naïve Huh-7.5 cells. Statistical significance of differences of means: n.s - not significant, * marginally significant (p≤0.1), ** significant (p≤0.05), *** highly significant (p≤0.01).
(TIF)

Figure S9 **HCV protease or polymerase inhibitors do not impede production of infectious particles in the transient assay.** Given drugs were applied to Jc1-transfected Huh-7.5 cells as outlined in Figure 1A. (A) HCV RNA replication in treated cells was determined by quantitative RT-PCR. (B) Release of HCV particles was determined by quantification of core protein levels in the culture fluid of the cells using a core-specific ELISA. (C) Infectivity of released particles was assessed using a limiting dilution assay. Data are shown as means +/− SD of three independent experiments.
(TIF)

Figure S10 **Subcellular localization of HCV core, ADRP, and GFP-PLA2G4A in the presence or absence of Py-2.** Stable cell lines ectopically expressing GFP-PLA2G4A were transfected with Jc1 and treated with Py-2 or were left untreated. Core protein expression was.
(TIF)

Figure S11 **Characterization of Huh-7.5-HA-ApoE cells.** (A) Endogenous ApoE expression in Huh7.5 cells was silenced using a lentiviral vector expressing an ApoE-specific shRNA. Subsequently, ApoE expression was restored by transduction of a mouse ApoE gene or an shRNA resistant, HA-tagged human ApoE gene by lentiviral gene transfer. ApoE and actin expression in the given cell lines was determined using antibodies for human ApoE and human actin. (B) Given cell lines were infected with the JcR-2a reporter virus carrying a *Renilla* luciferase gene [13]. RNA replication (left) and production of infectious particles (right) was monitored using luciferase assays (means +/− SEM are shown).
(TIF)
