# Supplementary figures and images for: MAP-Kinase Regulated Cytosolic Phospholipase A2 Activity Is Essential for Production of Infectious Hepatitis C Virus Particles
Source: PLoS Pathog. 2012 Jul 26;8(7):e1002829. doi: 10.1371/journal.ppat.1002829 (PMC3406102; doi:10.1371/journal.ppat.1002829)

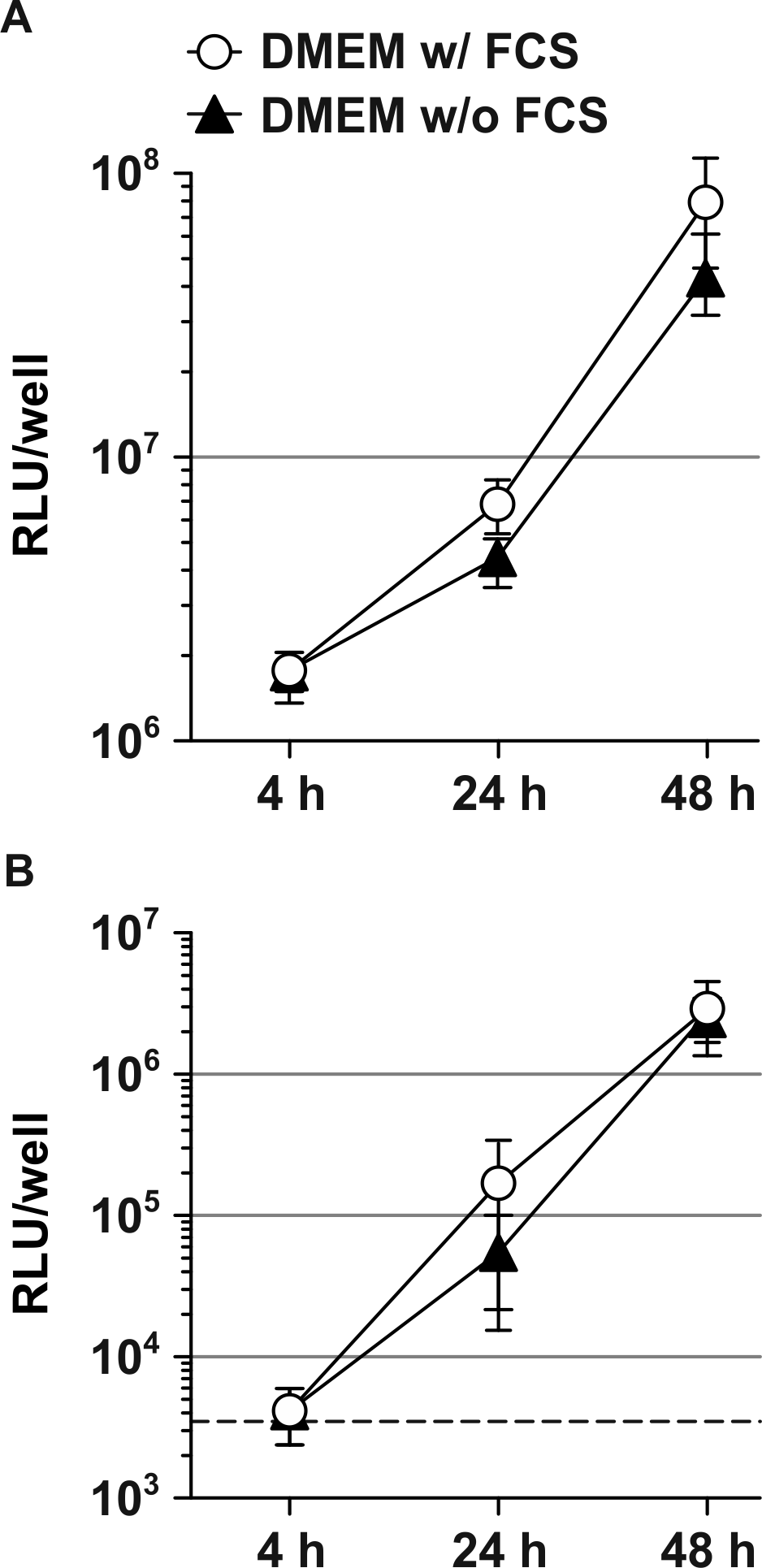

Supplement: Figure S1 — HCV RNA replication and virus production in Huh-7.5 cells cultured in the presence or absence of FCS. (A) Cells were transfected with Luc-Jc1. Four hours later medium was changed to culture fluid with or without 10% FCS. Cells and culture fluids were collected at given time points and RNA replication was determined by luciferase assays. (B) For detection of infectious particles, naïve Huh-7.5 cells were inoculated with the cell free supernatants harvested at the indicated time points. Luciferase activity in the inoculated cells was measured 72 h later. Data are shown as means +/− SD of three independent experiments. The dotted line represents background luciferase activity in mock infected cells. (TIF) [file ppat.1002829.s001.tif]

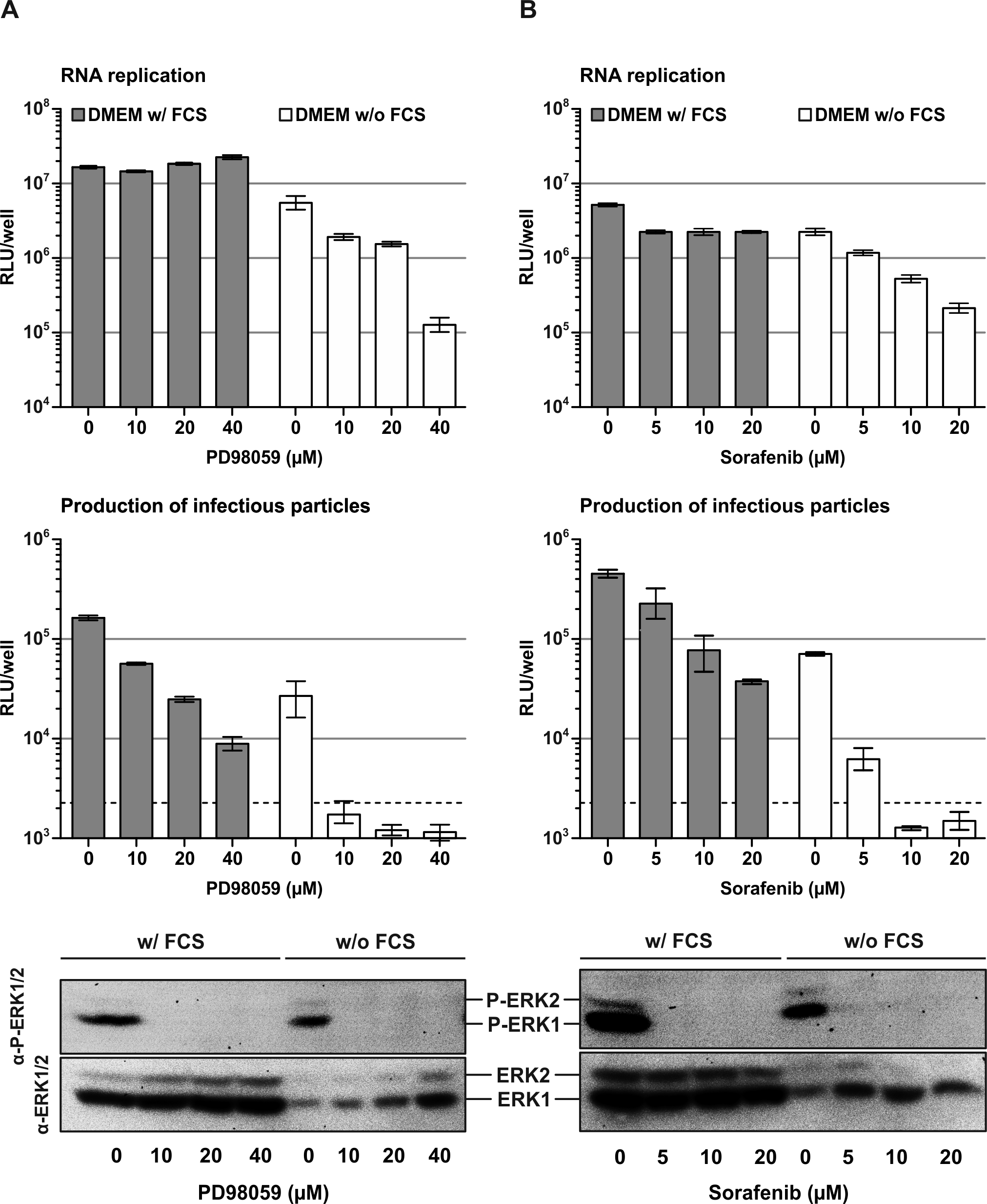

Supplement: Figure S2 — The MAPK/ERK inhibitors Sorafenib and PD98059 inhibit production of infectious HCV. (A, B) Cells were treated with the inhibitors as outlined in Figure 1A. HCV RNA replication in cells was measured by using a luciferase reporter assays (top panels). The release of infectious particles was determined by inoculation of naïve cells with culture fluids collected at 48 hpt and determination of luciferase activity in cells 72 h after inoculation (middle panels). Data are shown as means +/− SD of three independent experiments (the dotted line represents background luciferase activity in mock infected cells). The bottom panels display ERK1/2 expression and phosphorylation in Luc-Jc1 transfected and inhibitor treated cells. ERK proteins were detected as described in Figure 1. (TIF) [file ppat.1002829.s002.tif]

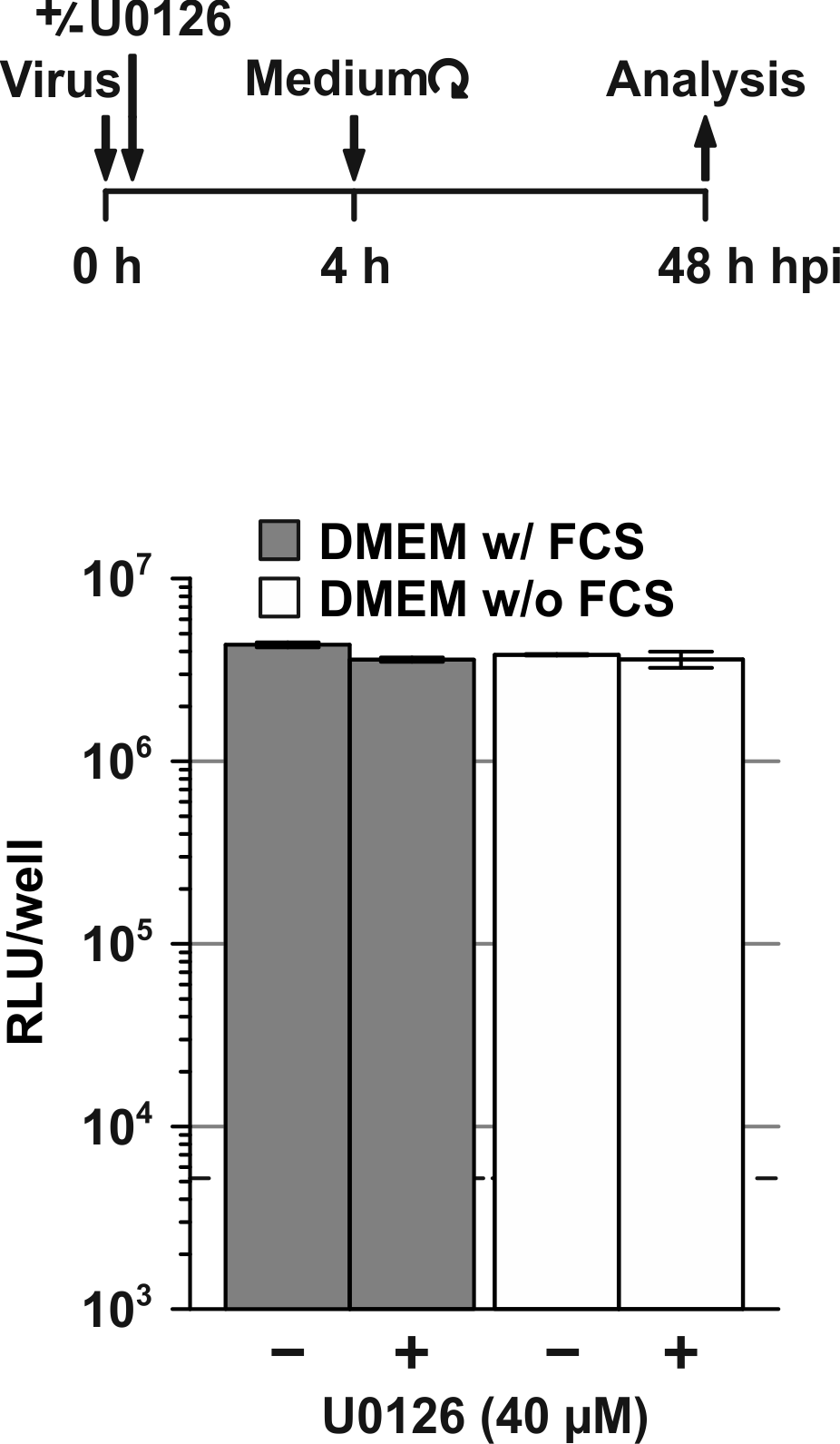

Supplement: Figure S3 — Influence of MAPK/ERK inhibitor U0126 on HCV cell entry. Luc-Jc1 particles prepared in the presence or absence of FCS were supplemented with the given dose of U0126 or left untreated. Virus suspensions were incubated with Huh-7.5 cells for 4 h at 37°C. Subsequently, unbound particles as well as the inhibitors were removed and cells were cultured in FCS-containing culture fluid until the analysis of HCV infection 72 h later. Data are shown as means +/− SD of three independent experiments. (TIF) [file ppat.1002829.s003.tif]

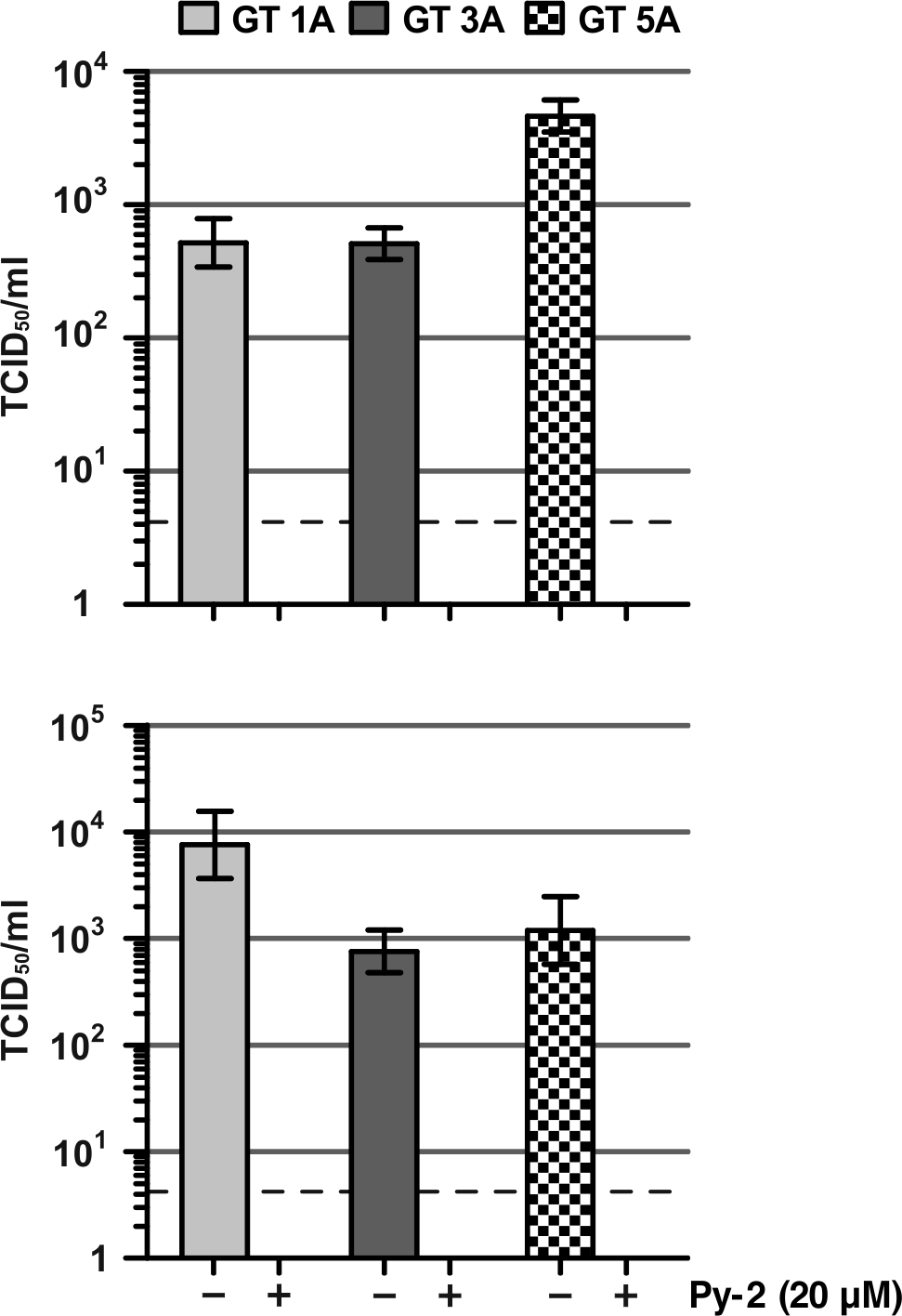

Supplement: Figure S4 — Py-2 impedes production of infectious HCV across different HCV genotypes. Cells were transfected with indicated chimeric HCV genomes encoding structural proteins of genotype 1a, 3a or 5a, and subsequently treated with Py-2 as described in Figure 1A. Production of infectious progeny was quantified using a limiting dilution assay. Two independent experiments are shown in the two panels. Mean values of six replicates +/− SD of the replicates are given. (TIF) [file ppat.1002829.s004.tif]

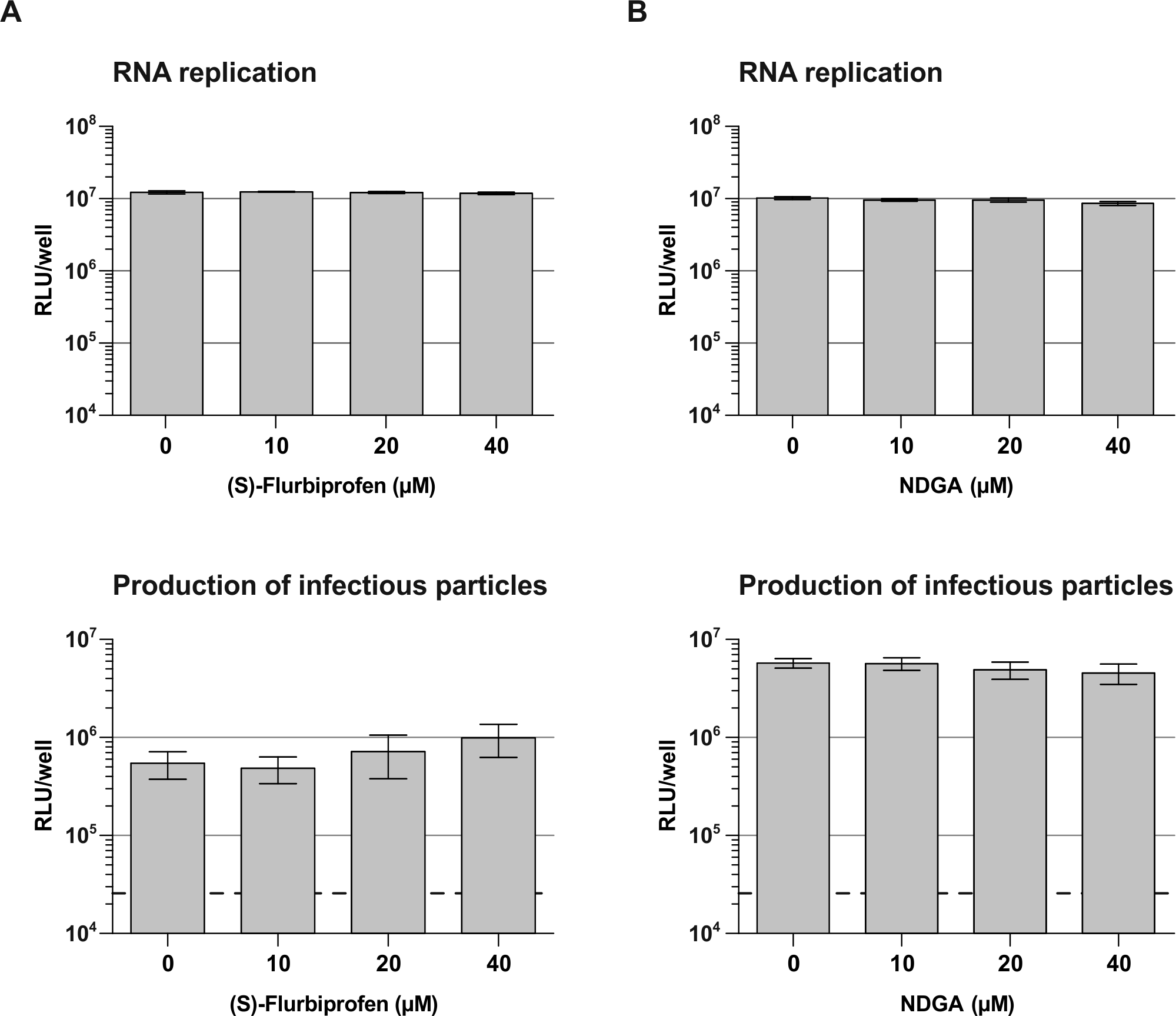

Supplement: Figure S5 — Blockade of arachidonic acid metabolism by inhibition of cyclooxygenases and lipoxygenases does not impede production of infectious HCV. Luc-Jc1 transfected Huh-7.5 cells were treated with given doses of (S)-Flurbiprofen (A) or NDGA (B) as outlined in Figure 1A. RNA replication in transfected cells and release of infectious particles was determined by luciferase asssays. Data are shown as means +/− SD of three independent experiments (the dotted line represents background luciferase activity in mock infected cells). (TIF) [file ppat.1002829.s005.tif]

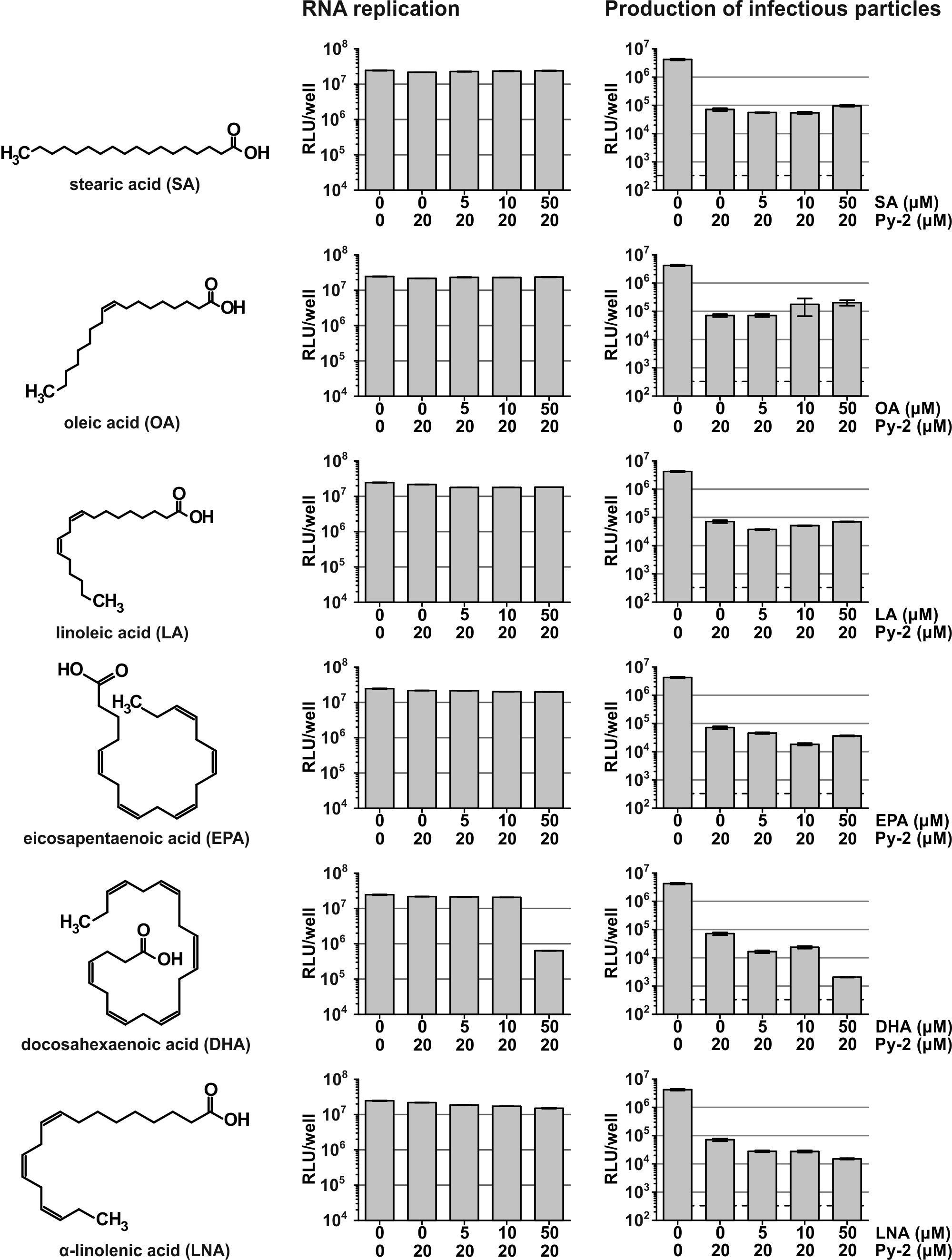

Supplement: Figure S6 — Fatty acids with varying degree of unsaturation are unable to restore virus production in Py-2-treated Huh-7.5 cells. Luc-Jc1-transfected cells were loaded with given lipids 32 hpt and subsequently subjected to the Py-2 inhibition assay outlined in Figure 1A. HCV RNA replication and virus production was determined by luciferase assays in cells treated with different fatty acids Data are shown as means +/− SD of three independent experiments (the dotted line represents background luciferase activity in mock infected cells). (TIF) [file ppat.1002829.s006.tif]

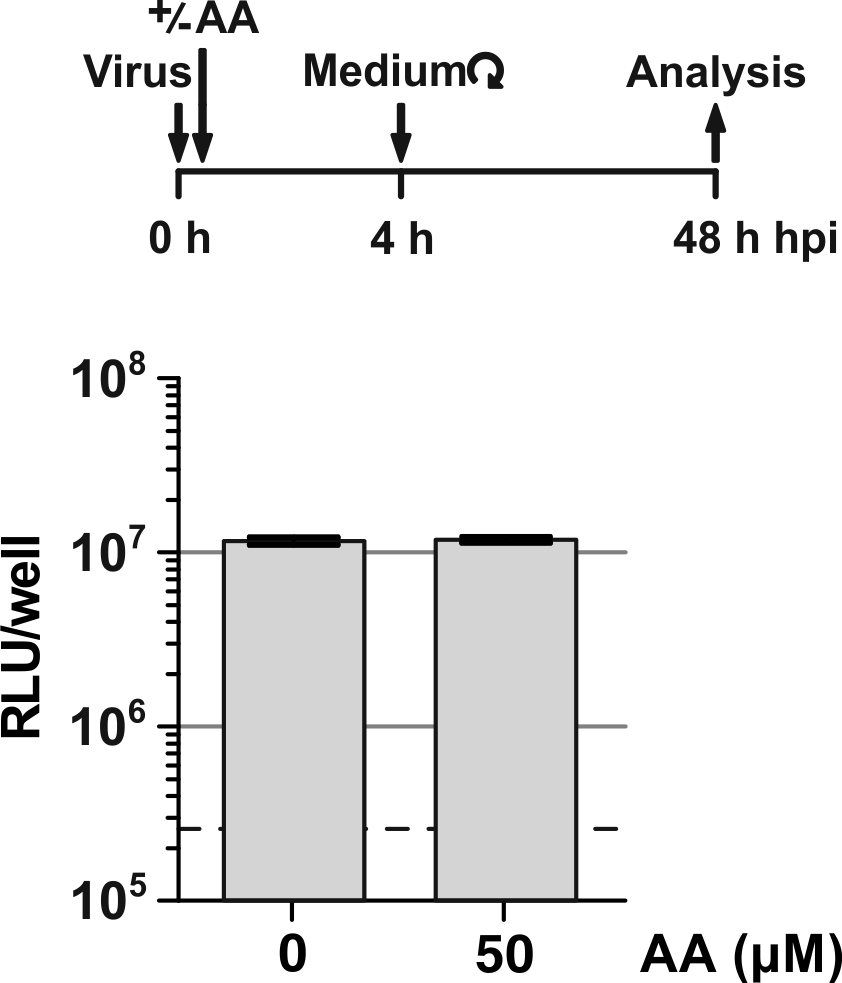

Supplement: Figure S7 — Arachidonic acid does not increase HCV cell entry. Luc-Jc1 particles were supplemented with AA or left untreated. Virus suspensions were incubated with Huh-7.5 cells for 4 h at 37°C. Subsequently, unbound particles as well as the inhibitors were removed and cells were cultured in FCS-containing culture fluid until the analysis of HCV infection 72 h later. Data are shown as means +/− SD of three independent experiments. (TIF) [file ppat.1002829.s007.tif]

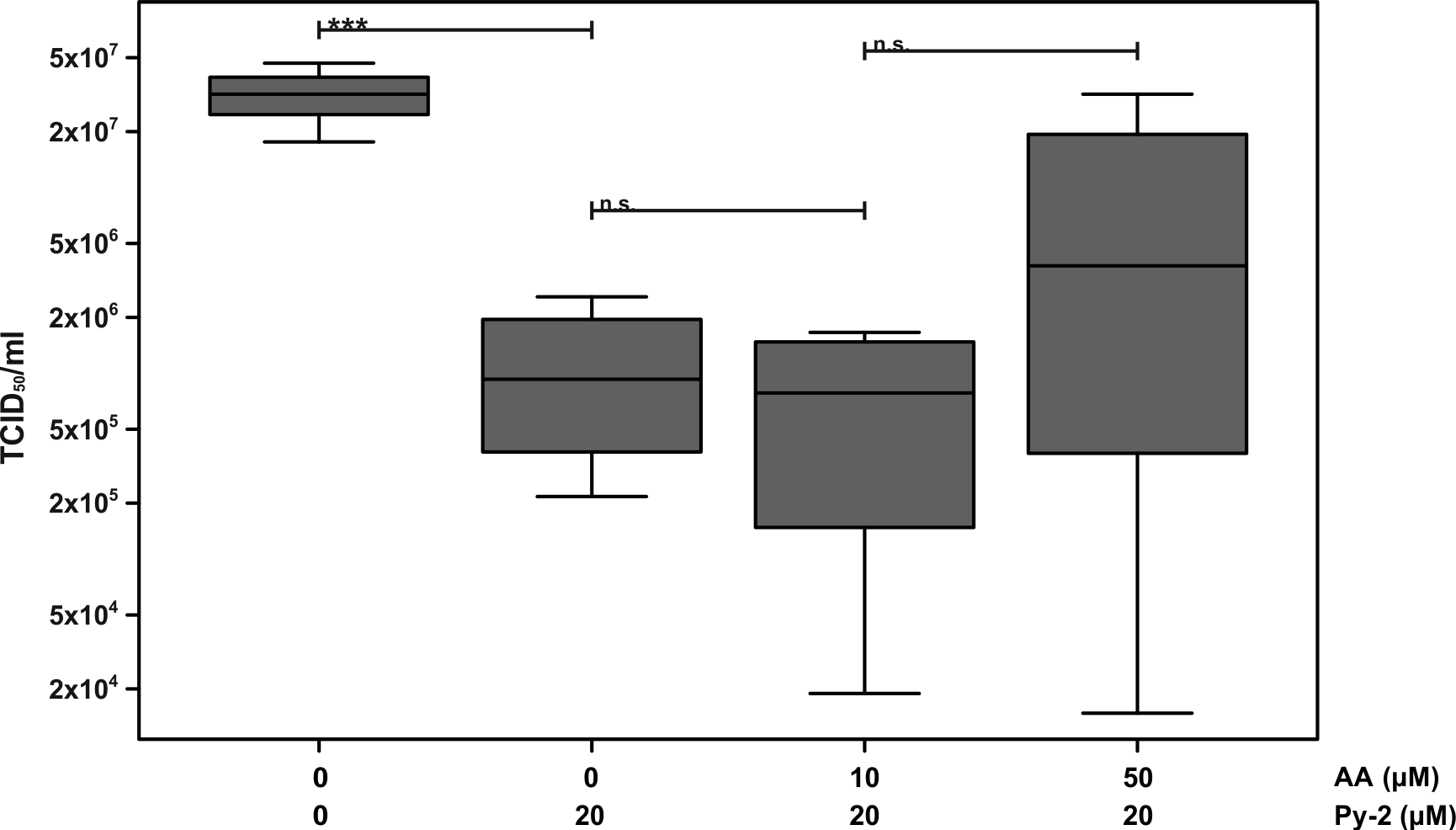

Supplement: Figure S8 — Influence of AA production of infectious DENV particles in the presence or absence of Py-2. Cells were transfected with a DENV RNA and treated as described in Figure 1A. Infectivity of released particles was determined by inoculation of naïve Huh-7.5 cells. Statistical significance of differences of means: n.s - not significant, * marginally significant (p≤0.1), ** significant (p≤0.05), *** highly significant (p≤0.01). (TIF) [file ppat.1002829.s008.tif]

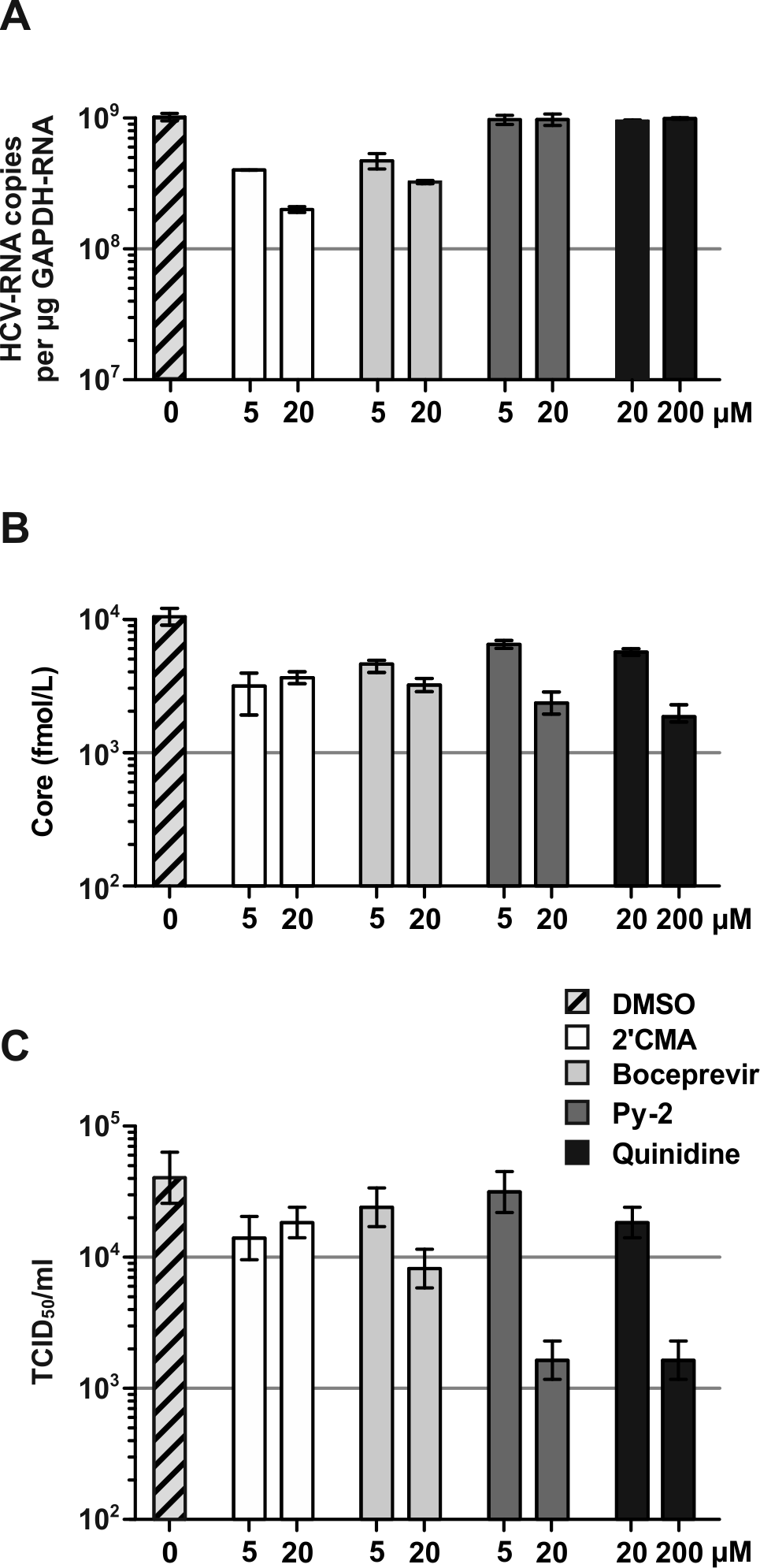

Supplement: Figure S9 — HCV protease or polymerase inhibitors do not impede production of infectious particles in the transient assay. Given drugs were applied to Jc1-transfected Huh-7.5 cells as outlined in Figure 1A. (A) HCV RNA replication in treated cells was determined by quantitative RT-PCR. (B) Release of HCV particles was determined by quantification of core protein levels in the culture fluid of the cells using a core-specific ELISA. (C) Infectivity of released particles was assessed using a limiting dilution assay. Data are shown as means +/− SD of three independent experiments. (TIF) [file ppat.1002829.s009.tif]

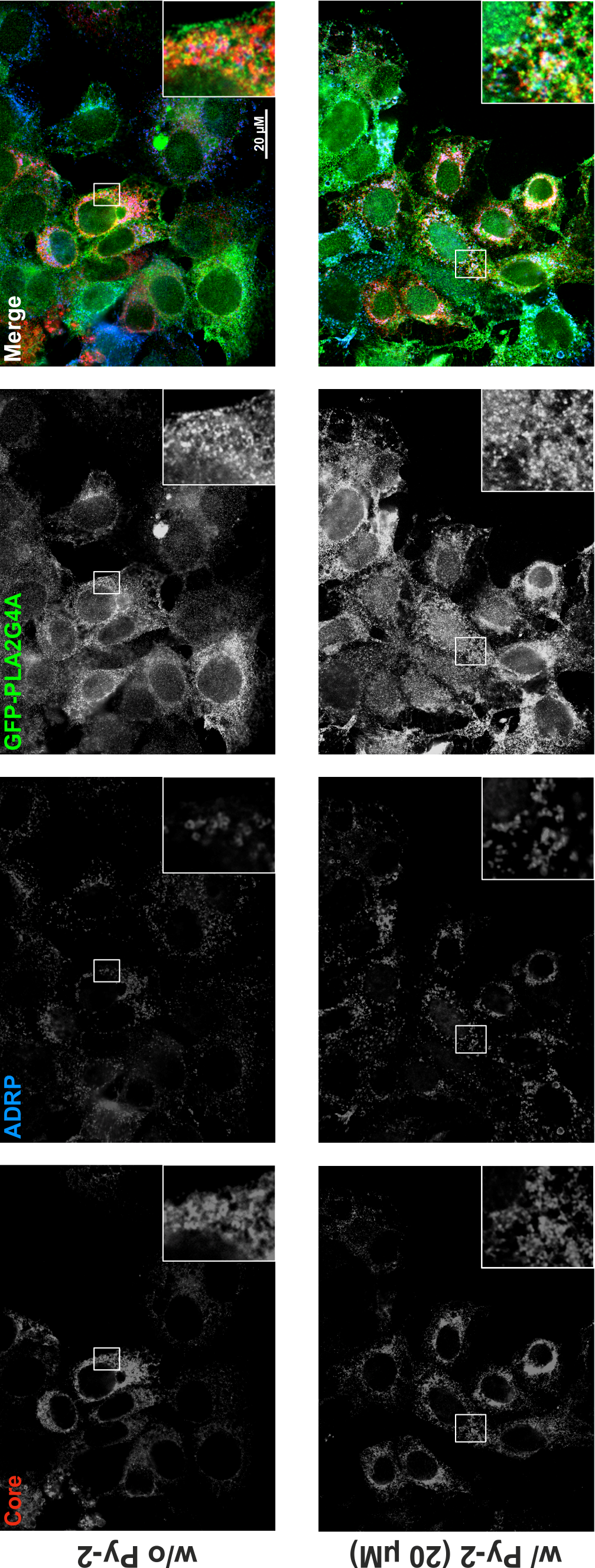

Supplement: Figure S10 — Subcellular localization of HCV core, ADRP, and GFP-PLA2G4A in the presence or absence of Py-2. Stable cell lines ectopically expressing GFP-PLA2G4A were transfected with Jc1 and treated with Py-2 or were left untreated. Core protein expression was. (TIF) [file ppat.1002829.s010.tif]

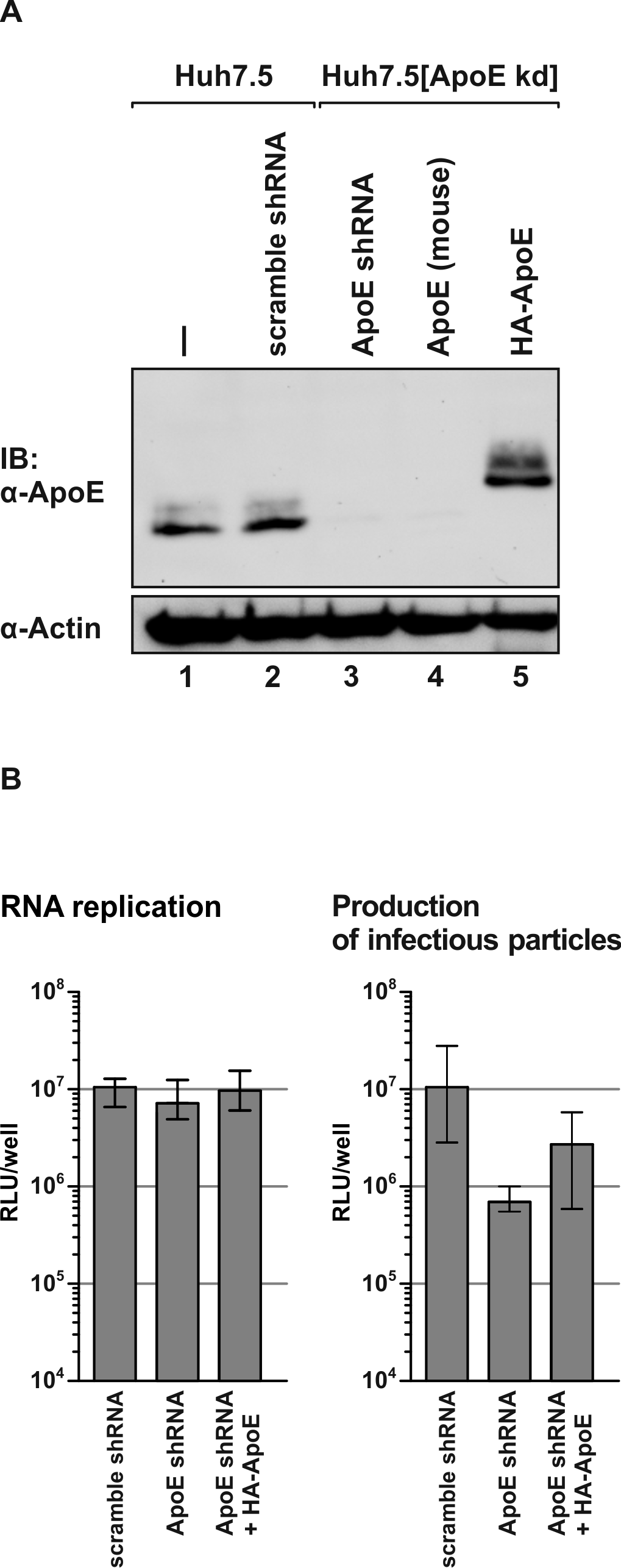

Supplement: Figure S11 — Characterization of Huh-7.5-HA-ApoE cells. (A) Endogenous ApoE expression in Huh7.5 cells was silenced using a lentiviral vector expressing an ApoE-specific shRNA. Subsequently, ApoE expression was restored by transduction of a mouse ApoE gene or an shRNA resistant, HA-tagged human ApoE gene by lentiviral gene transfer. ApoE and actin expression in the given cell lines was determined using antibodies for human ApoE and human actin. (B) Given cell lines were infected with the JcR-2a reporter virus carrying a Renilla luciferase gene [13]. RNA replication (left) and production of infectious particles (right) was monitored using luciferase assays (means +/− SEM are shown). (TIF) [file ppat.1002829.s011.tif]
